# A review of the techno-economic potential and environmental impact analysis through life cycle assessment of parabolic trough collector towards the contribution of sustainable energy

**DOI:** 10.1016/j.heliyon.2023.e17626

**Published:** 2023-06-26

**Authors:** Prashant Saini, Shweta Singh, Priyanka Kajal, Atul Dhar, Nikhil Khot, M.E. Mohamed, Satvasheel Powar

**Affiliations:** a School of Engineering, Indian Institute of Technology Mandi, Mandi, Himachal Pradesh, 175005, India; b United Nations Industrial Development Organization, UN House, 55, Lodhi Estate, New Delhi, 110003, India; c Chemistry Department, Alexandria University, Egypt; d Alamein International University, Alamein City, Matrouh Governorate, Egypt; e School of Technology and Business Studies, Energy Technology, Dalarna University, Falun, 791 31, Sweden

**Keywords:** Solar parabolic trough collector, Geometric analysis, Thermal analysis, Heat transfer enhancement, Heat transfer fluid, Life cycle assessment

## Abstract

Parabolic trough collectors (P.T.Cs) are efficient solar energy harvesting devices utilized in various industries, for instance, space heating, solar cooling, solar drying, pasteurization, sterilization, electricity generation, process heat, solar cooking, and many other applications. However, their usage is limited as the high capital and operating costs; according to the International Renewable Energy Agency's 2020 report, the global weighted average levelized cost of electricity (L.C.O.E) for P.T.Cs was 0.185 $/kWh in 2018. This work analyses the economic, technical, and environmental potential of sustainable energy to increase the use of P.T.Cs in different sectors. To study how self-weight, heat loss, and wind velocity affect P.T.C performance, prototype testing, and wind flow analysis were used. Although P.T.Cs outperform in capacity factor, gross-to-net conversion, and annual energy production, improving their overall efficiency is crucial in reducing total energy production costs. Wire coils, discs, and twisted tape-type inserts can enhance their performance by increasing turbulence and heat transfer area. Improving the system's overall efficiency by enhancing the functioning and operation of individual components will also help decrease total energy production costs. The aim is to minimize the L.C.O.E associated with a P.T.C in order to enhance its economic viability for an extended period. When the nanofluid-oriented P.T.C was included in the conventional P.T.C workings, there was a decrease in the L.C.O.E by 1%. Of all the technologies available, ocean, geothermal, and C.S.P parabolic trough plants generate lower amounts of waste and harmful gases, with average emissions of 2.39%, 2.23%, and 2.16%, respectively, throughout their lifespan. For solar-only and non-hybrid thermal energy storage plants, the range of greenhouse gas emissions is between 20 and 34 kgCO_2_ equivalents per megawatt-hour. Coal, natural gas steam turbines, nuclear power plants, bioenergy, solar PV, geothermal, concentrated solar power, hydropower reservoir, hydropower river, ocean, and wind power plants all release greenhouse gases at rates of 1022, 587.5, 110.5, 633, 111, 48, 41, 82.5, 7.5, 12.5, and 41.5 gCO_2_-e/kWh, respectively. This information is useful to compare the environmental effect of various energy sources and help us to choose cleaner, more sustainable options for the production of electricity. The ongoing advancements and future scope of P.T.Cs could potentially make them more economically viable for domestic, commercial, and industrial applications.

## 1 Introduction

An adequate supply of energy is an important source for a country's economic development. Energy in various forms, such as coal, natural gas, oil, nuclear energy, hydropower, and others, is required to meet household and industrial needs. According to the Bureau of Energy Efficiency's 2021 survey, energy consumption has increased over the years and will continue to rise in the coming years. According to the Bureau of Energy Efficiency 2021, oil consumption in developed and developing countries is at an all-time high. Oil consumption in France, Canada, United States, the United Kingdom, China, India, and Japan was 914.3 million tonnes, 96.4 million tonnes, 94.2 million tonnes, 76.8 million tonnes, 275.2 million tonnes, 113.3 million tonnes, and 248.7 million tonnes, respectively [1].

Similarly, coal consumption in France, Canada, United States, the United Kingdom, China, India, and Japan reached an all-time high of 573.9 million tonnes, 31.0 million tonnes, 12.4 million tonnes, 39.1 million tonnes, 799.7 million tonnes, 185.3 million tonnes, and 112.2 million tonnes, respectively [1]. Increasing trends in other energy resources have also been reported. The increased use of energy raises consumption and production costs, affecting the environment, investment, savings, and the operations of individuals and industries. In India, for example, the government determines the price of natural gas, which ranges from INR 5 to INR 15 per cubic meter, depending on the amount of energy allocated to consumers. On the other hand, electricity prices range from INR 150 to INR 300 per kVA, depending on the industrial end-use sector [1].

**Fig. 1:**
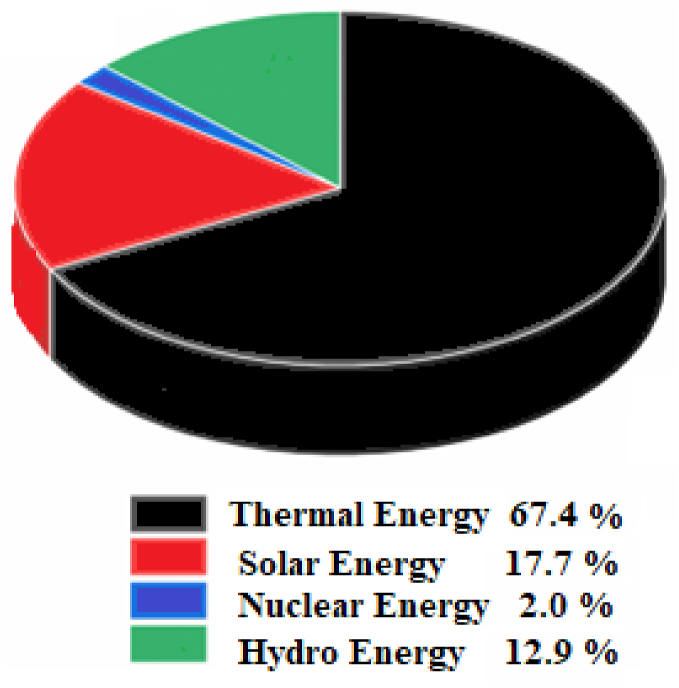
Power generation in India from different E.Ss [3].

As a result, more renewable energy sources (E.Ss) must be introduced to increase energy production propensities and reduce production costs to meet the diverse energy needs of households and industries. Thus, in the current and future scenarios, renewable (solar, biomass, water, wind, geothermal) and non-renewable (natural gas, coal, nuclear, oil) energy resources together can meet the increasing energy demand for the growing population and industrial needs. In the last two decades, there has been an increase in the use of renewable energy in power generation to meet energy demands and fill the demand gap created by non-renewable energy resources in prominent applications such as industrial desalination and heating, refrigeration, and electrical power generation [2]. According to India's Central Electricity Authority report of 2022, the country requires 132,507 MW of peak power, but only 128,083 MW is available [3]. Fig. 1 depicts the contribution of various E.Ss, like thermal, nuclear, solar, and hydropower, to meet the energy demand of India.

Solar energy (SE) is one of the most significant contributors in terms of renewable E.Ss for producing energy for use in many applications. Solar-based technologies are quickly becoming one of the most successfully engineered technologies, paving the way for future endeavours [4]. Solar power is derived from the sun, which is the most abundant source of energy on the planet and is available in all parts of the world. There is still a need for an energy-efficient system to extract maximum energy from diverse renewable E.Ss despite the many technological advances that have been made in this area. SE is a prominent and abundant E.S for generating electrical power [5]. As progress toward harnessing the sun's power continues, it will soon be the most E.S used for the betterment of humanity and the planet [6]. SE can be captured and used to generate heat (using solar thermal collectors) or directly converted into electricity (using solar photovoltaics). Photovoltaic modules directly convert SE into electricity. However, this harvesting technique is less efficient than solar thermal, which is significantly more efficient. According to the United Nations Sustainable Development Goal 9 (Industry, Innovation, and Infrastructure) report, heating accounts for a significant portion of energy consumption in industries. Process heating in industries necessitates temperatures ranging from 50°C to 250°C. We can easily replace this temperature requirement of industries using renewable E.Ss by harvesting SE via solar thermal.

Different solar collectors have engaged in thermal use, such as flat plate collectors, evacuated tube collectors, P.T.Cs, point focus collectors, and concentration collectors. For the temperature range of 50°C–250°C, the P.T.C is an efficient instrument that extracts SE and utilizes it for different purposes, including electricity generation, process heat, space heating, solar cooling, solar cooking, pasteurization, sterilization, and many more. For example, P.T.C is used in an air heating system to dry marine foodstuff, farm products, and textiles. The solar air heating system is also used in collaboration with heat storage systems such as the thermal energy storage system (T.E.S.S), which stores the energy by changing its phase from solid to liquid. In this process, non-paraffins, hydrated salts, and fatty acids store thermal energy. T.E.S.S helps in reducing electric energy consumption during the peak period by 32% and the off period by 90% [7]**.** It encourages using a renewable E.S and reduces costs related to non-renewable resources. Thus, P.T.C is highly economically viable and reduces the power production cost significantly using SE.

The collection of SE to convert into a useable medium of energy is performed with the help of developing technology termed Concentrating Solar Power, which involves adjusting the aperture area of the surface responsible for collecting the sun rays. It is then transferred to the area where the heat is transferred to the fluid that helps store the heat in a storage unit and later can be utilized for power generation [8,9]. P.T.Cs are majorly employed for power generation applications [10]. Various advancements in the design and development of P.T.Cs have made them attractive candidates for industrial applications such as industrial process heating and institutional cooking [11].

The solar collector's performance is the most crucial criterion for power generation plants using SE as their primary source. One of the prominent thoughts works for exploiting SE on a larger scale [12]. Various solar collector modules are designed for low and high-temperature solar plants to optimize the solar collector's thermo-economical optimization. Among solar collectors, P.T.Cs are one of the prominent collector modules because of their superior performance and diversified applicability [13]. P.T.Cs are largely employed in Concentrated Solar Power (C.S.P) plants, which command high and low-temperature heat. The C.S.P plant typically consists of a large area incorporating single-axis P.T.C to help under certain constraints during solar radiation's unavailability [14].

C.S.Ps utilize SE in the form of heat energy, designed to minimize the design and implementation expenses while improving the thermal efficiency of solar P.T.Cs [15]. In C.S.Ps, Heat Transfer Fluids (H.T.Fs) such as oil and water are irrelevant as they are easily evaporated and superheated. Additionally, using dematerialized water in C.S.Ps is found to be economical. Compared with oil, water usage was found to be reasonable and affordable as water is non-toxic and non-flammable. Climate change, declining oil reserves, and political unrest in traditional oil-producing countries are just a few of the factors that have contributed to the growing interest in solar P.T.Cs with improved performance. Solar P.T.Cs are widely available and have made great strides toward completely replacing fossil fuels in the energy sector. Taking a cue from P.T.C's prominence in solar power plants, researchers have now shifted their focus to developing advanced solar P.T.Cs and making P.T.C-based solar power plants affordable and cost-effective [16].

P.T.C system is used in desalination projects to obtain low-cost fresh water. The distiller attached to the P.T.C consists of a solar concentrator and absorber cavity that helps absorb solar power in large quantities. The simple thermal insulation and wind protection installed in the cavity absorber help increase freshwater productivity by two folds by efficiently using direction and position during the day. It saves the costs related to non-renewable resources and contributes toward the green and sustainable source of freshwater production [17].

The P.T.C system induced SE for refrigeration and air conditioning by making use of dehumidification properties. The different workings pairs, such as silica gel–water and zeolite–water, carbon–methanol increase the capacity of a solar-driven adsorption system that reduces the temperature and provides a cooling effect without using a heat pipe [18]. P.T.C reduces carbon footprint levels, saves non-renewable energy, and provides a cost-efficient refrigeration and air conditioning process.

P.T.C is also used for industrial purposes such as cooking, sterilization, drying, degreasing, pasteurization, and others to use SE efficiently [19]. The use of P.T.C could also be recognized in power plants, where it is used to extract SE and convert it into electricity. For instance, P.T.C generates 400° C of heat when used with synthetic oil as a heat-transferring fluid. It can be used directly for steam generation and enhances the enactment of solar power plants [20]. Thus, it can be said that with the help of P.T.C, there is the generation of high energy, a reduction in carbon footprint, efficient use of a renewable source of energy, and a decrease in the use of non-renewable resources, which makes it an economically viable tool. A new type of P.T.C was made with improved characteristics. It uses mineral oil nanofluid as its working fluid and has multiple types of receiver tubes. This model successfully achieved an increased efficiency by 11% [21]. Apart from mineral oil Nanofluid, molten salt is also used in various P.T.C models to increase the P.T.C's efficiency even further [22].

Ionic liquids known as molten salts can also be employed as a heat transfer medium in C.S.P plants. A C.S.P concentrates sunlight through mirrors or lenses onto a receiver containing molten salt, which absorbs the heat and transmits it to a power unit, where it is used to produce electricity. The low-temperature molten salt is again returned to the receiver for further heating [23]. Because of its high specific heat capacity, molten salts can take in and hold a lot of heat energy. As a result, C.S.Ps can continue to produce power even when the sun is not shining, such as during cloudy or night-time conditions. The stability of molten salts at high temperatures is another benefit of employing them. Molten salts are an excellent heat transfer fluid for C.S.P systems that run at high temperatures since they can sustain temperatures of up to 600°C. Molten salts are a safer option than other heat transfer fluids like oil because they are non-toxic and inflammable as well. Despite the benefits of using molten salts, there are significant drawbacks to their application in C.S.P systems. The price of the salt itself, which can be pricey, is one difficulty. Another issue is the corrosion of the system's materials, which might be brought on by the molten salt's chemical composition and high temperatures.

Researchers have discussed the facts related to the use of P.T.C in existing literature, but more focus should also be given to its economic potential. Therefore, the current review article analyzes the technical, economic, and environmental potential of sustainable energy contribution to reduce the gap in the existing literature. The article also explores the fundamentals of P.T.C, its modeling, and its life cycle assessment (L.C.A) by focusing on its economic aspects and determining its performance and economic potential using different analysis procedures such as optical analysis, heat transfer analysis, and others. The environmental consequences of switching to P.T.C systems are also reviewed.

## 2 P.T.C system

**Fig. 2:**
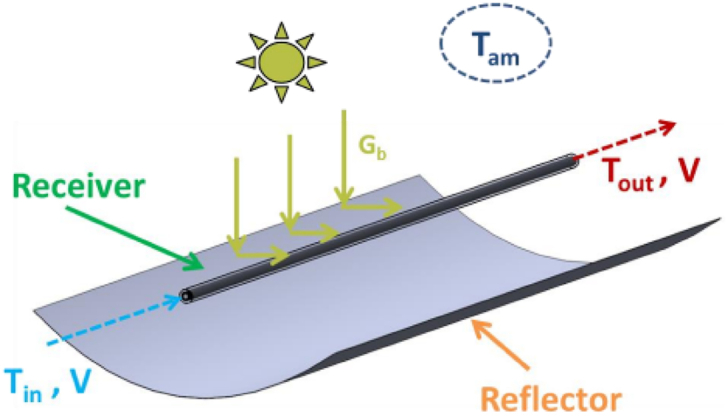
A typical P.T.C module (Creative Commons Attribution (CC BY) license) (http://creativecommons.org/licenses/by/4.0/).

In early 1983, the P.T.C design was suggested that put forth an ideology that the curved trays as a collector to gain as much energy as possible from the sun's rays. The design of the collector fitted with the reflector surface in a curved shape was inspired by the 1913 model of Frank Schuman's design for irrigation panels in the field [24]. According to Bellos et al. [25], the conventional P.T.C comprises different parts, such as a linear tubular receiver, concentrator, and metallic support structure. Fig. 2 represents the general model of P.T.C, which tracks the sun using a single-axis mechanism so that solar rays are amassed in the collector aperture. P.T.Cs track the sun's movement to ensure a continuous focus on the linear receiver [26]. In the conventional model, the P.T.C is placed on the North-South axis to track the sun rays coming from the East-West direction. It helps in the maximum utilization of SE, resulting in the optimization of solar potential. Moreover, to enhance the efficiency of P.T.C, several modules are placed in linear series at a close distance of 10 m to generate more energy. The fundamental arithmetic of the conventional P.T.C in the segment of parabolic shape geometry is expressed by equation (1) [25].

(1) $$y=\frac{x^{2}}{4f}$$

The rim angle *(φ*_*r*_*)* is calculated using the aperture (*W*), and the focal distance (*f*) is represented with the help of equation (2) [25].

(2) $$\varphi_{r}=\arctan\left[\frac{8\cdot \frac{f}{w}}{16\cdot {\left(\frac{f}{w}\right)}^{2}-1}\right]$$

Equation (3) expresses the product of Width (*W*) and Length (*L*) as the total collector aperture (*A*_*a*_) [25].

(3) $$A_{a}=W\cdot L$$

The outer area of the tube and the absorber area (*A*_ro_) is represented through equation (4) [25].

(4) $$A_{ro}=\pi \cdot D_{ro}\cdot L$$

Equation (5) represents the definition of the geometrical concentration ratio (*C*) as the ratio of collector aperture (*A*_*a*_) to absorber area (*A*_*ro*_) [25].

(5) $$C=\frac{A_{a}}{A_{ro}}$$

**Fig. 3:**
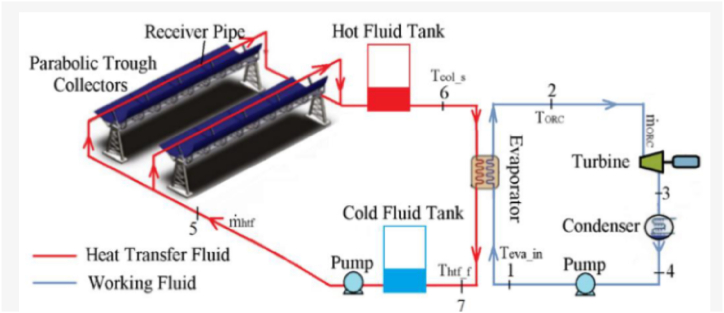
Illustration of P.T.C-based solar power plant (Creative Commons Attribution International License (CC BY)) (https://creativecommons.org/licenses/by/4.0/).

Identifying the potential factors responsible for enhancing the performance of the P.T.Cs and evaluating performance efficiency in P.T.C power plants requires meticulous knowledge of the factors affecting the optical performance of solar power plants. The radiant energy from the sun is absorbed by a P.T.C, which then transforms it into useable thermal energy in the heat transfer fluid (H.T.F) that flows through the collector's receiving tube. The energy efficiency of H.T.F can be determined after evaluating the thermophysical properties of the fluid. The operating temperature of P.T.C is about 400°C, and the most used H.T.F is synthetic oil [27]. Parabolic troughs use synthetic oil as a coolant to decrease the temperature of the superheated steam. Hence, a heat exchanger, usually composed of a mixture containing oil and water or steam, is required between the solar P.T.C collector and the power block. This arrangement is regarded as H.T.F technology, which is abundantly adopted in P.T.C-based thermal plants [28]. The selection of H.T.F comprises all necessary mathematical equations required to analyze different expressions related to energy balancing, dependent on environmental constraints and the optical characteristics of the collector receiver [29]. Kumaresan et al. [30] presented an experimental evaluation to determine the performance of a solar P.T.C integrated with a storage unit. The study uses Therminol 55 as H.T.F. It was observed from the analysis that the temperature of H.T.F was increased when the experimental evaluation was performed for a day. The performance efficiency of the P.T.C was found to be increased during the time duration of 8.00 and 9.00 h. P.T.C achieved the instantaneous peak efficiency was 62.5% at 12.00 h. The schematic of a P.T.C solar power plant is illustrated in Fig. 3 [31]. P.T.Cs consist of parabolic trough-shaped mirrors, where the radiation from the sunlight is reflected on the solar receiver tube [32], as shown in Fig. 3 below. The absorbed radiant energy heats the circulating H.T.F, which flows through the receiver. After heating, the H.T.F is aggregated and passed to the power block, traversing through cascaded heat exchangers and generating superheated steam at elevated temperatures. This steam passes through a steam turbine, which converts the steam into electrical power [33]. The optical analysis, geometric analysis, computational fluid dynamics (CFD) analysis, and performance enhancement techniques of P.T.C are discussed in further sections.

## 3 Performance analysis of the P.T.C

P.T.C system is increasingly used in solar power plants because of their simple design and cost-effective structure. However, the cost of the P.T.C system increases if there is an error (random and non-random) in the solar extraction or heat generation process. Under such conditions, optical analysis, geometrical analysis, CFD analysis, etc., helps identify the errors and eliminate them by estimating the energy ratio between the receiver and incident ray to the collector aperture.

### 3.1 Geometrical analysis of P.T.C

The design and development of solar P.T.C are performed based on the requirement and type of application [34,35]. Hence, multiple methods are involved in the design of P.T.C. While most of the P.T.C systems are designed with a supporting structure, reflector, receiver, tracking system, H.T.F, and thermal storage device.

#### 3.1.1 Structure of P.T.C

**Fig. 4:**
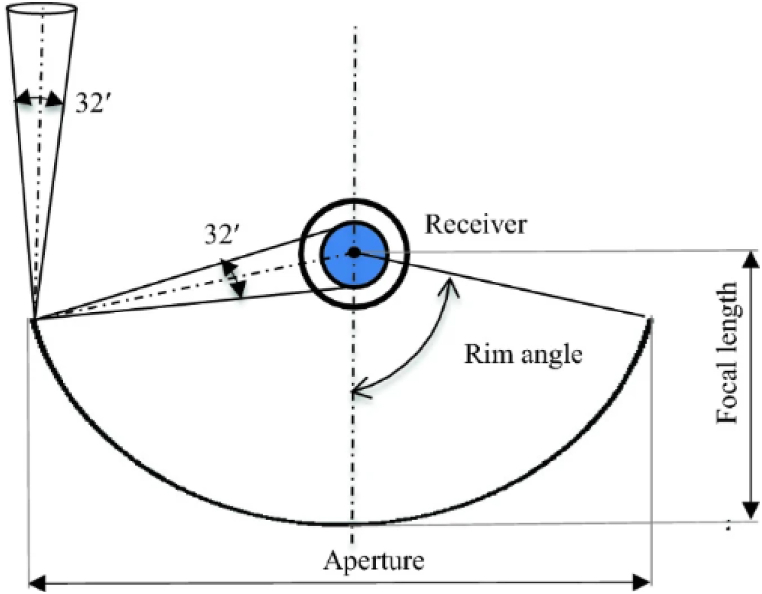
Basic design parameters of parabolic trough concentrator (License number 5536360332722).

The basic structure of the solar P.T.C is shown in Fig. 4 [36].

The basic design of the P.T.C is shown in Fig. 4. P.T.C consists of a linear imagining concentrator and a parabolic trough-shaped reflector, which reflects the sun's radiation and transfers the radiation into a receiver tube positioned at the parabola's focal line [36]. The three significant components constituting the design of P.T.C are.

(i) Ratio of geometric concentration
(ii) Acceptance angle
(iii) Rim angle

The geometric concentration ratio is a prominent parameter among acceptance and rim angle. The concentration ratio constitutes the fraction of the collector aperture region to the area of the receiver. The concentration ratio is defined as shown in equation (6) [36].

(6) $$Concentration\;ratio=\frac{1}{\sin\;\theta_{s}}$$

*θ*_*s*_ is defined as the half-angle subtended by the sun; *θ*_*s*_ = 0.27.

The geometrical parameters of the solar P.T.C are rim angle, the width of the aperture, focal length, and trough length. Various research has been conducted to optimize the solar P.T.C parameters to make them adaptable for a wide range of applications and different geographical environments. If the rim angle is very less, the aperture area of the P.T.C gets extended, resulting in a higher focal length [37]. This increases the complexity of the structure of P.T.C and decreases its cost-effectiveness because of the increased design cost. Whereas, if there is an increase in the rim angle, there will be a reduction in the aperture area and focal length. A lower rim angle also leads to the parabolic radius elevation, which elevates the mirror cost. The solar P.T.C can absorb solar radiation and transfer it to the working fluid or H.T.F. The H.T.F can be thermal oil, air, or organic solvents. As P.T.C possesses a higher heat absorption rate, it is one of the most widely researched solar collectors compared to flat plate collectors with reflectors [38,39].

### 3.2 Optical analysis of P.T.C

The optical analysis determines the system's performance by examining the interaction between the charged particles of matter and light. It helps in ascertaining the absorbance and intensity of the solar wavelengths. By conducting an optical analysis of P.T.C, the ratio of energy absorbed by the received against each incident ray collected on the aperture is determined. As a result, the efficiency of the P.T.C system is determined by the errors associated with P.T.C surfaces. For example, random errors occur because of changes in the width of sun rays, slope errors, and wind loading, while non-random errors occur because of misalignment of angle, inappropriate reflector profile, and others. These errors impact the performance and economic viability of P.T.C.

Guven et al. [40] analyzed that optical efficiency is the photo-thermal conversion procedure in which the energy ratio is determined between the absorbed heat collector element (H.C.E) and the aperture collector. It is denoted by *η*_o_ and includes the functioning of other elements such as glass envelope (τ), absorber surface (α), and mirror, and H.C.E interaction (γ) is used to obtain the energy incident levels. It is expressed by equation (7) [40].

(7) $$\eta_{ο}\left(\theta =0\right)=\rho \tau \alpha \gamma$$

The implications related to incidence angle are determined with the help of the incidence-angle modifier. It also helps to determine the correlation between the modifier and incidence angle so that variations in optical features can be determined. It is expressed through equation (8) [40].

(8) $$K\left(\theta \right)=\frac{\eta_{o}\left(\theta \right)}{\eta_{o}\left(\theta =0\right)}$$

Equation (9) determines the end loss factor when all the H.C.Es are placed at equal lengths symmetrically, and when the length of H.C.E is more than the length of l, the end loss factor is modified by equation (10) [41].

(9) $$\Gamma =1-\frac{f}{l}\left(1+\frac{w_{2}^{a}}{48f^{2}}\right)\tan\;\theta$$

(10) $$\Gamma =1+\frac{r}{l}-\frac{f}{l}\left(1+\frac{w_{2}^{a}}{48f^{2}}\right)\tan\;\theta$$

**Fig. 5:**
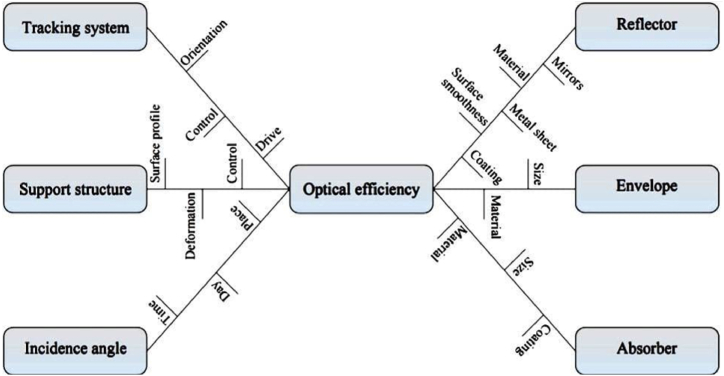
Parameters affecting optical efficiency [41].

According to Guven et al. [42], the performance of the optical design of P.T.C is impacted by different factors such as the width of the sun's rays, the incidence angle of the sun's rays, physical attributes of the heat collector, errors, operating procedures, and others. Mokheimer et al. [43] analyzed the implication of the P.T.C elements based on optical performance, which is represented in Fig. 5.

As per Fig. 5, it can be said that different factors impact the optical performance of the P.T.Cs, such as the tracking system, support structure, reflector, glass envelope, absorber, and incidence angle. The other sub-factors, such as size, coating, mirrors, materials, place, control system, etc., also impact the optical efficiency of P.T.C.

#### 3.2.1 The support structure, incident angle, and tracking system

P.T.C structure gets distorted under heavy wind load, which will decrease the optical efficiency of the overall system. But nowadays, the glass mirrors of the P.T.Cs are designed to withstand the maximum wind load of up to 37 m/s [44]. The heavy supporting structure results in a better performance against the wind load, too, but it results in a rise in the overall initial cost of the P.T.C system and increases the payback period of system. A less rigid structure may reduce the overall cost, but the error due to wind load must be higher. Many researchers have tried using different torque tubes, such as box-type structures and cylindrical hollow tubes [45,46]. Fu et al. [47] simulated the P.T.C to determine the wind structure interaction and optimized their new model to withstand the higher wind load. So, Fu et al. [47] reduced the overall weight of the collector by up to 5.8% compared to earlier models. The tracking system is another factor affecting the overall performance of the P.T.C system. A good tracking system has less tracking error of - 4 milliradians–4 milliradians. As the incident angle of solar radiation increases, the tracking error plays an important role in the circumferential flux distribution on the absorber tube [48]. The tracking system depends upon other factors, such as orientation, control, and drive. All these factors should also be taken care of during the setup of the tracking system.

#### 3.2.2 Reflector, envelope, and absorber tube

The reflecting surface of the mirror reflector is coated with silver, followed by copper. Some layers of paint are applied to it to increase the durability of the highly polished mirror surface, which has a reflectivity of around 94.5%. The highly reflective aluminium sheets only have a reflectivity of around 88%. Commercially used mirror reflectors of 4 mm thickness are AGC solar mirror, RIOGlass, and for 1 mm, its RONDAGlass [[49], [50], [51]]. The mirror reflectors are intended to be used for the higher temperature range. Aluminium reflectors of thicknesses 0.3 mm and 0.4 mm are used only for the low-temperature range ALMECO [52] and ALMIRA [53]. The Anti-reflective coating has been applied in optical industries to enhance the overall quality of the images and reduce glare. In the case of solar application, the borosilicate glass has a good transmissivity property, which makes this glass to be used as concentric with an absorber tube of P.T.C. The anti-reflective coating on this borosilicate glass enhances its transmissivity property by around 92%–96% [54]. Mcleod and Hecht modeled the anti-reflective coatings and their operation principle [55,56]. The refractive index of borosilicate glass is 1.47, and when it is coated lower RI coating of MgF_2,_ it reduces the refractive index to 1.37. Optical efficiency is defined as the ratio of the total energy absorbed by the absorber tube to the total energy incident on the aperture surface. So, it all depends upon the collector's geometry and the materials' optical properties [57]. The use of selective surface on the absorber tube has started since 1970 and continues. So, from the extensive review from 1970, it is observed that the coating of Ni–NiO has the largest absorptivity of around 0.96 (ϵ_100_ = 0.10), and the coating of different materials such as Ni, Ti, Ag, and Cu has a minimum emissivity of around 0.01–0.02 (α = 0.71–0.80). So, much scope for improvement in the emissivity property of the different materials persists [[58], [59], [60]].

### 3.3 Thermal analysis of P.T.C

The thermal efficiency of a P.T.C is a measure of how effectively it converts solar radiation into thermal energy. The thermal efficiency can be calculated using equation (11) [61].

(11) $$\eta_{th}=\frac{Q_{u}}{Q_{s}}$$

where *Q*_*u*_ is the useful heat, and *Q*_*s*_ is the solar radiation incident on the collector. The *Q*_*u*_ can be determined using equation (12) [62].

(12) $$Q_{u}=mC_{p}\Delta T$$

where *m* is the mass flow rate of the fluid, *C*_*p*_ is the specific heat capacity of the fluid, and *ΔT* is the temperature difference between the inlet and outlet of the collector. The heat absorbed by the H.T.F is estimated by equation (13) [62].

(13) $$Q=mC_{p}\left(T_{out}-T_{in}\right)$$

where m is the mass flow rate of the H.T.F, *C*_*p*_ is the specific heat capacity of the H.T.F, *T*_*out*_ is the outlet temperature of the H.T.F, and *T*_*in*_ is the inlet temperature of the H.T.F.

The design of the collector, the type and quality of the H.T.F, and the operating conditions/parameters of the system are some additional variables that might impact a P.T.C's thermal efficiency in addition to these calculations. To accurately anticipate the performance of a P.T.C system, a thorough thermal study should consider each of these elements.

**Fig. 6:**
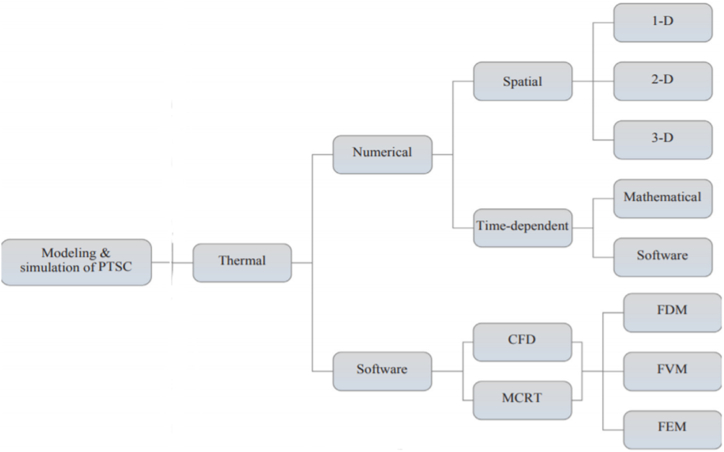
Methodology pursued modeling and simulation of P.T.Cs.

Jeter et al. [63] analyzed that thermal analysis of P.T.Cs helps estimate the temperature at the surface level, determine the efficiency of the solar absorber, and evaluate the thermal loss of H.C.E. A thermal model is developed to determine the temperature profiles and heat flux in thermal analysis. Fig. 6 represents the thermal model used in the P.T.C efficiency estimation process. The accurate thermal performance of H.C.E is determined by equation (14), as it helps determine the local heat flux allocation along the circumference of the H.C.E [63].

(14) $$LCR=\frac{q^{\prime \prime }}{{Ι}_{b,n}}$$

Equation (14) also helps determine the normal incident radiation beam levels and ascertains the performance of geometrical features of P.T.C, such as border position, meditation proportion, optical inaccuracy, and frequency position. Gong et al. [64] analyzed that a one-dimensional (1-D) theoretical model is used to determine the vacuum in H.C.E under ambient conditions.

Bendt et al. [65] examined that ray tracing is an approach used to analyze the optical performance of the P.T.Cs. The ray-tracing technique is helpful in systems that have multiple surfaces and unsuitable Newtonian and Gaussian imaging equations. In the ray-tracing model, the sun rays are traced with the help of optical elements and propagate light in different mediums as per their specific features. The ray-tracing approach provides detailed information about the optical features of the P.T.C. However, the major limitation of the ray-tracing model is that it does not provide information related to functional relationships. Fraidenraich et al. [66] analyzed that the assessment of overall heat loss from H.C.E is determined with the help of the thermal loss coefficient, which is expressed as U_L_. It is a simple analysis technique that helps predict the thermal loss coefficient by analyzing multiple numerical iterations. Duffie et al. [67] analyzed that the thermal loss coefficient is more efficient than numerical assessment as it is less time-consuming and tedious. It also helps in analyzing the thermal efficacy of P.T.C by equation (15) [67].

(15) $$\eta_{t}=\frac{\int A_{a}\left[\eta_{o}\left(\theta \right)I_{b}-U_{L}\left(T_{HCE}-T_{a}\right)\right]dt}{A_{a}\int I_{b}dt}$$

**Fig. 7:**
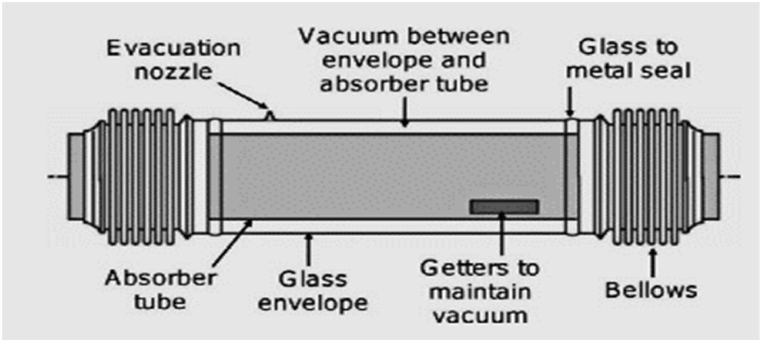
Schematic of a typical heat-collecting element (Creative Commons Attribution International License (CC BY)).

Benoit et al. [68] examined that H.C.E forms an essential part of P.T.C that is made up of an absorber tube in an envelope, as shown in Fig. 7 [69].

The heat flow within the absorber is determined with the help of forced convection in a single or two-phase manner. Amongst both phases, the single-phase is known as a more efficient method as it does not require a change of H.T.F during the operation.

Edenburn et al. [70] examined that in the single-phase steady flow, the temperature of the P.T.C system is maintained, which increases the reliability of operation and controllability levels. It is an easy process compared to the two-phase that effectively helps forecast the fluid function of P.T.C. It includes energy balance equations (16), (17), (18) to conserve energy at every H.C.E surface [70].

(16) $$Q_{absorbed\;solar}=Q_{residual}+Q_{radiation,abs}+Q_{\sup\;port,bellow}$$

(17) $$Q_{beam\;radiation}=Q_{optical}+Q_{convection}+Q_{radiation,env}$$

(18) $$Q_{heat\;loss}=Q_{convection}+Q_{radiation,env}+Q_{\sup\;port,bellow}$$

Ouagued et al. [71] analyzed that most of the P.T.Cs function as per transient conditions owing to the heating up of the collectors from start to shut down time every day. It includes analyzing heat transfer fluid (H.T.F) temperature, mass flow rate, and solar radiation to evaluate the performance of dynamics present in P.T.C.

Enciso Contreras et al. [72] analyzed that the flow features in direct steam generation (DSG) are more complicated than in the single-phase flow assessment process. The major reason behind instability is disturbances in the flow of SE because of clouds or changes in weather conditions. Under such conditions, the transient modeling provides more precised information related to solar field prediction as the predictions made by single-phase, two-phase, or dynamic changes in the weather conditions impact dry streams. It leads to fluctuations in the outlet temperature, thermal stability issues, and poor operation of the desired flow regime. You et al. [73] examined that the operational complexity in the DSG system is different from the oil-based P.T.C plants. As a result, the P.T.C system must be executed with the help of different modeling tools such as TRNSYS and RELAP so that its efficiency is improved and an increase in the overall efficiency reduces the cost of electricity generation.

By assessing the pressure loss and effective thermal input in solar P.T.Cs, which have a temperature of about 5800 K, one can ascertain the exergetic performance of the solar P.T.C. These two effects demonstrate the production ability of P.T.Cs in electric power generation. In our situation, we ignore the pressure loss to streamline our calculations and concentrate on the effective thermal input in solar P.T.Cs, which is the primary element affecting the performance and efficiency of P.T.Cs.

The formation of entropy is given by equation (19) [74].

(19) $$S_{gen}={\left(\frac{dS}{dt}\right)}_{CV}-\sum_{i=0}^{n}\frac{Q_{i}}{T_{i}}-\sum_{0}m_{0}S_{0}\geq 0$$

The absorber tube's exergy rate is determined by using the Petela model, as shown by equation (20) [75,76].

(20) *Ex*_*u*_ = *mC*_*pf*_[(*T*_*out*_-*T*_*in*_)-*T*_*amb*_ln(*T*_*out*_/*T*_*in*_)]

In addition, the absorbed solar radiation exergy by the solar receiver tube and reflector is given the Petela model, as shown in equation (21) [77,78].

(21) *Ex*_*a*_ = *A*_*ap*_*I*_*D*_[1=(1/3) (*T*_*amb*_/*T*_*S*_)^4^-(4*T*_*amb*_/3*T*_*S*_)]

### 3.4 CFD analysis of P.T.C

A computer-based simulation technique called computational fluid dynamics (CFD) is used to study and resolve issues involving fluid flows, heat transport, and other related processes. It is a potent tool that permits scientists and engineers to research and improve the way fluids behave in a variety of situations, including turbulence, pressure fluctuations, and heat gradients. Numerous industries, including aerospace, automotive, energy, and environmental engineering, heavily rely on CFD [[79], [80], [81], [82], [83], [84]].

In CFD, mathematical models are employed to represent the behavior of fluids, which are then solved via numerical methods. The basic steps involved in CFD simulations are:

1. Pre-processing: To do this, the geometry must be established, the boundary conditions must be specified, and suitable numerical methods for the simulation must be chosen.
2. Solving: The equations describing the behaviour of fluids are numerically solved in this step using a variety of numerical techniques, including finite element analysis, the finite volume method, and the finite difference approach.
3. Post-processing: To do this, the simulation results must be analyzed and then visualized using a variety of tools, including graphs, animations, and contour plots.

According to Al-Ansary et al. [79], CFD is an effective tool for resolving fluid-related problems that lack evaluation-based solutions. As a result, it is frequently used to simulate, evaluate, and optimize P.T.C engineering ideas. The use of modern computer techniques in CFD helps to resolve problems with fluid mechanics and heat transfer design. The development of computer-based computational capabilities makes it possible to solve challenging issues involving arbitrary geometries and physics in a constrained amount of time. Additionally, the advancement of numerical algorithms has decreased the expense of calculation. Significant insights on replicating data, flow fields, and design processes can be gained via CFD research. It is also known to yield better findings than experimental and analytical approaches, and it allows for the study of the P.T.C system's practical constraints.

With the aid of CFD, ANSYS software assists in the analysis of the governing equations about the numerical modeling of P.T.C [80]. When it comes to P.T.C, CFD is also utilized to assess the thermal-hydraulic performance of H.C.E and look at the state of the wind in the system's surrounding area. As a result, CFD offers numerical solutions to equations relating to continuity, energy balance, and mass balance. After determining whether the flow is laminar or turbulent, equations are proposed in CFD. As a result, problems in continuity and energy balance are identified, which enhances P.T.C's effectiveness. By effectively utilizing renewable energy and utilizing less non-renewable E.Ss, it aids in the generation of high power.

Kumar & Reddy et al. [81] analyzed that in the CFD process, the actual boundary conditions are used to predict the efficacy of P.T.C. Amongst the several boundary conditions, ascertaining heat flux is an important aspect as it is related to the H.C.E absorber performance analysis. As a result, it helps attain precised outcomes related to H.C.E thermal analysis by estimating the actual heat flux profile. CFD analysis includes a coupling technique that is based on the MCRT technique. It helps determine the heat flux allocation more efficiently than the longitudinal angle process. Behar et al. [82] stated that heat transfer mechanisms could also be included in the CFD analysis process as it helps analyze the annulus conditions, especially in the case of commercial H.C.Es. It helped to determine the heat transfer radiations between the glass envelope and absorber tube using the surface-to-surface (S2S) radiation model. Mwesigye et al. [83] examined that the discrete ordinates (DO) radiation model is also used with CFD to determine the vacuum present in the air. If the annulus space is not estimated, the other two aspects, natural convection heat transfer and radiation heat transfer, are included for analysis. In the case of natural convection heat transfer, there is the determination of annulus space by using energy and Navier-Stokes equations. On the other hand, the radiation heat transfer is treated by using energy and Navier-Stokes equations to evacuate H.C.E.

Mathew et al. [84] analyzed that CFD software ANSYS Fluent is highly helpful in modeling the geometric structure of P.T.C. It helps locate the exact position of the sector patch by using a solar calculator. As a result, there is the determination of the transformation angle of rotation for different seasons (summer and winter) and performing ray tracing activities. The ray-tracing technique is helpful in systems that have multiple surfaces and unsuitable Newtonian and Gaussian imaging equations. In the ray-tracing model, the sun rays are traced with the help of optical elements and propagate light in different mediums as per their specific features. The ray-tracing approach provides detailed information about the optical features of the P.T.C. However, the major limitation of the ray-tracing model is that it does not provide information related to functional relationships.

Zhang et al. [85] analyzed that CFD is applied in the P.T.C system to oscillate flow within the pulse tube cryocooler, which comprises a compressor and heat exchanger. It includes overall heat loss determination from H.C.E with the help of the thermal loss coefficient, which is expressed as U_L_. It is a simple analysis technique that helps predict the thermal loss coefficient by analyzing multiple numerical iterations. Thus, by using CFD analysis, the determination of oscillating flow and H.C.E helps enhance the performance of the P.T.C system. Therefore, it can be said that with the help of P.T.C, there is the generation of high energy, a reduction in carbon footprint, efficient use of a renewable source of energy, and a decrease in the use of non-renewable resources, which makes it an economically viable tool.

### 3.5 Performance enhancement techniques

The most recent developments in P.T.C technology are concentrated on enhancing productivity, lowering expenses, and boosting the system's dependability. The technology is projected to see considerable breakthroughs with further research and development in these fields, becoming a more significant part of the renewable energy landscape.

#### 3.5.1 Thermal energy storage (T.E.S)

P.T.C systems that use C.S.P frequently include thermal energy storage (T.E.S). This is so that C.S.P systems, such as parabolic troughs, can produce electricity by turning steam generated by solar heat into electricity through a turbine. However, sunlight is sporadic, and the amount of solar radiation that the parabolic troughs can capture might change depending on things like the time of day and the weather. Therefore, T.E.S is required to guarantee a constant flow of electricity [86,87].

The P.T.C produces heat during the day. T.E.S devices use this heat to generate electricity during cloudy weather and nighttime. Creating new materials for storage systems and optimizing storage operation procedures are just two recent examples of research aimed at enhancing the effectiveness and affordability of T.E.S [88].

Using novel materials that have a higher heat capacity and can store more heat for longer periods is one way to improve T.E.S capacity. For instance, some researchers have looked into the usage of materials that go through a phase change and absorb and release heat (e.g., from solid to liquid). T.E.S is more effective and affordable, could result from phase-change materials since they have been found to have a larger heat capacity than conventional storage materials like water or molten salt [89,90].

Optimizing the performance of T.E.S devices involves a different strategy. This may entail adjusting the charging and discharging of the storage system based on the weather and the demand for electricity using sophisticated control systems. For instance, researchers have looked into using machine learning and predictive algorithms to optimize the performance of T.E.S systems based on real-time weather data and electricity demand forecasts [91]. In addition to these strategies, some researchers are also looking into the usage of hybrid T.E.S systems, which mix various types of storage materials to increase the system's overall efficiency and economic viability. For instance, a hybrid thermal storage system could combine phase-change materials with molten salt to offer both short-term and long-term storage capabilities [92].

In general, it is essential to increase the effectiveness and affordability of T.E.S if we want to continue developing and deploying concentrated solar power systems, such as P.T.Cs. It is anticipated that more studies in this field will result in substantial technological breakthroughs, making it a more vital part of the renewable energy landscape.

#### 3.5.2 Hybrid systems

Hybrid systems that combine P.T.C with other renewable energy technologies, such as photovoltaics, wind power, or geothermal energy, are being investigated to maximize energy production and boost system reliability. These hybrid energy systems can aid in addressing some of the problems that come with relying solely on renewable E.Ss, such as intermittent nature and unpredictability in power output [93].

Adding photovoltaic (PV) systems to P.T.C is one method of hybridizing them. PV systems, which can be utilized to supplement the electricity generation of P.T.Cs, produce electricity directly from sunlight using semiconducting materials. This strategy may help to boost the system's overall energy output because PV systems can produce power when there is little sunshine, and the P.T.Cs are less efficient [94].

P.T.Cs and wind power systems working together is another strategy. In times of low sunlight, wind power systems can be utilized to enhance P.T.Cs' ability to produce electricity by collecting the kinetic energy of the wind. This strategy could contribute to increasing the system's overall energy output because wind power systems can provide electricity when P.T.Cs are less efficient owing to weather [95].

Some researchers are also investigating the usage of hybrid systems, which pair P.T.Cs with geothermal energy, in addition to these strategies. When there is little sunshine, geothermal energy systems can be utilized to complement P.T.Cs' ability to produce power by drawing on the heat that is stored in the earth's crust. This strategy can assist in increasing the system's overall energy output because geothermal energy systems can produce electricity when P.T.Cs are less effective owing to environmental factors [96].

Overall, hybrid energy systems incorporating P.T.Cs and other renewable energy technologies have the potential to boost energy output, boost system dependability, and minimize the system's overall environmental effect. Continued study in this field is expected to result in large technological breakthroughs, making it a more significant part of the renewable energy landscape.

#### 3.5.3 Control systems

Advanced control systems, including the use of machine learning and predictive algorithms, are being developed to optimize the operation of P.T.Cs. These systems will increase performance and decrease downtime. For P.T.Cs, cutting-edge control methods are being created to boost their effectiveness and dependability. These control systems employ various technologies, like machine learning and predictive algorithms, to enhance the collectors' performance and decrease downtime.

Using predictive algorithms to estimate solar radiation and change the orientation of the mirrors accordingly is one method of enhancing the performance of the P.T.C. This could enhance the system's effectiveness and maximize the capture of solar radiation. Additionally, predictive algorithms can be utilized to forecast weather conditions and modify the operation of the collectors, accordingly reducing downtime and enhancing system dependability [97].

Another strategy is to optimize the performance of the collectors using machine learning techniques for previous data. Machine learning algorithms can analyze large volumes of operational data to find trends and improve system performance. For instance, using historical weather and electricity demand data, machine learning algorithms can be utilized to optimize the charging and discharging of T.E.S systems [98].

Along with these strategies, several researchers are also looking into the application of cutting-edge control systems to enhance the upkeep and repair of P.T.Cs. These control systems can monitor and diagnose system issues using sensors and other technologies, and they may then automatically start maintenance or repair procedures as necessary [99].

Overall, in the subject of P.T.C, advanced control systems represent a significant area of study. These technologies have the potential to dramatically increase the technology's efficiency and dependability, lowering costs and enhancing the performance of the renewable energy system as a whole. More studies in this field are anticipated to result in substantial technological breakthroughs, making it a more vital part of the renewable energy landscape.

#### 3.5.4 New materials

To increase P.T.C's effectiveness and lower costs, researchers have been looking at the usage of novel materials in recent years. One approach is to use nanostructured materials, which have unique properties that can enhance the absorption and reflection of sunlight [100]. To boost the absorption of solar radiation, researchers have studied the usage of nanowires, which can trap light [101]. This enhanced absorption may result in higher temperatures and greater efficiency in P.T.Cs.

Utilizing selective coatings on the trough surfaces is an alternative strategy. Selective coatings are made to reflect a small amount of solar light while absorbing a large portion of it. Lowering the amount of solar radiation lost as heat, selective absorption, and reflection can improve the collector's efficiency. It has been demonstrated that some selective coatings can improve the effectiveness of P.T.Cs [102].

Utilizing innovative materials can assist in cutting costs in addition to increasing efficiency. For instance, some researchers have considered using less expensive iron-based alloys for the receiver tubes than those used in commercial systems. Other researchers have considered using lightweight components, including carbon fiber, to lighten and make the construction of the supporting structure of the P.T.C cheap [103].

Several issues need to be resolved, even if the introduction of novel materials holds out a lot of potential for increasing effectiveness and lowering the price of P.T.Cs. For instance, compared to the materials now employed in commercial systems, some of these novel materials might be less enduring or heat resistant. Furthermore, the manufacturing procedures for these novel materials can be more difficult or expensive than those used now.

Verma et al. [104] analyzed that the determination of the nanoparticle-laden fluid flow properties is essential to predict the performance of the P.T.C system. The efficiency of P.T.C can be improved by bringing changes in the thermophysical features of H.T.F or by modifying the structure of conventional H.T.Fs with thermally conductive materials. Modified Maxwell's model is applied to spherical inclusions in the form of equation (22) [104].

(22) k_nf_ = [k_f_+(n-1)k_f_-(n-1)*φ*(k_f_-k_p_)]/[k_p_+(n+1)k_f_+*φ*(k_f_-k_p_)]

On the other hand, the density of nanofluids *(****ρ***_*nf*_) can be determined by equation (23) [34].

(23) $$\rho_{nf}=\left(1-\varphi \right)\rho_{b}+\varphi \left(\rho_{p}\right)$$

The specific heat capacity of nanofluids *(C*_*nf*_*)* can be estimated using equation (24) [34].

(24) $$C_{nf}=\frac{\left(1-\varphi \right)\rho_{b}C_{b}+\varphi \rho_{p}C_{p}}{\rho_{nf}}$$

The viscosity of nanofluids (*μ*_*nf*_) can be determined using equation (25) [34].

(25) $$\mu_{nf}=\mu_{b}\left(123\varphi^{2}+7.3\varphi +1\right)$$

where the subscript *(n*_*f*_*)* represents nanofluids, *(p)* refers to the nanoparticle, *(b)* is the base fluid, *μ* is the viscosity, and *φ* is the volume fraction of nanoparticles.

Nanofluids are a class of fluids that consist of suspended nanoparticles in a base fluid. These fluids exhibit unique thermal properties compared to their base fluids, such as increased thermal conductivity, which makes them attractive for use in various engineering applications, including P.T.C systems [34,104,105].

In P.T.C systems, a nanofluid's density significantly impacts its thermal characteristics and performance. The base fluid becomes denser due to the inclusion of nanoparticles, which causes higher thermal inertia and a slower response to temperature flocculation. This trait can contribute to fewer temperature changes inside the P.T.C system, improving system performance and stability [106]. Another thermal characteristic of a nanofluid that is crucial in evaluating how well it performs in P.T.C systems is its specific heat capacity. The specific heat capacity of the base fluid increases with the addition of nanoparticles, allowing for the storage of more thermal energy. As more thermal energy can be captured and used, this attribute can help the P.T.C system run more efficiently [106]. A third essential thermal characteristic of a nanofluid that affects how well it performs in P.T.C systems is viscosity. The base fluid becomes more viscous when nanoparticles are added, which may affect how quickly fluid moves through the system. However, the viscosity improvement can also boost the fluid's convective heat transfer coefficient, improving system performance and increasing thermal efficiency. The use of nanofluids in P.T.C systems has shown promising results in terms of improving their thermal performance. The enhanced specific heat capacity, density, thermal conductivity, and viscosity of nanofluids can help to increase the efficiency of P.T.C systems, leading to better energy utilization and reduced environmental impact [34,104].

Overall, the study of novel materials in the field of P.T.Cs is a fascinating one. The efficiency and cost-effectiveness of this technology are expected to improve significantly because of ongoing developments, making it a crucial part of the renewable energy landscape.

According to Giglio et al. [107], increasing the efficacy of P.T.C at low cost is a major concern among scholars, engineers, and technicians. To reduce this issue and increase optical efficiency, different novel designs have been developed by engineers. For example, FDM based solution method has been proposed to increase the optical efficiency of P.T.C. The novel design includes the formation of porous H.C.E with a partially insulated envelope. It increases the diameter of the envelope, because of which there is a low strike rate of solar radiation on the mirror. The design is based on a 1-D steady-state and transient approach that helps improve insulation conductivity and reduce heat loss by 40%. A study was conducted by Bader et al. [108] to propose a novel design to bring improvements in the performance of the P.T.C system. The novel design was based on the MCRT/FVM method in which an air-based tubular cavity is inserted in H.C.E. It is made up of a single-glazed configuration with higher efficacy than double-glazed solar radiation panels. As a result, there is an improvement in the flow rate leading to augmented collector thermal performance. It also benefitted in lowering the HFT mass flow rates and reduced the weight by 5 kg/s. It helped attain higher collector efficacy at 300°C compared to the double-glazed configuration that showed efficacy at 400°C. Other novel designs have also been introduced by researchers such as Xiao et al. [109], in the form of a V-cavity absorber, triangular cavity absorber, upper half insulated H.C.E, and others that helped in increasing the efficiency of the P.T.C system.

Gan et al. [110] examined that researchers have introduced several passive heat transfer improvement techniques to increase the performance of P.T.C. For example, the study conducted by Hegazy et al. [111] proposed an iteration-based solution method in which the absorber was modified by attaching longitudinal fins. On the other hand, the FVM-based model is proposed by Cheng et al. [112] in the form of trapezoidal, triangular, porous square, and circular inserts. The technique is based on a 3-D steady-state and RNG k–ε turbulence model that helps in reducing heat loss from the H.C.E. MCRT/FVM-based resolution technique was proposed as Unilateral longitudinal vortexes. The solution was based on a 3-D steady-state and DO radiation model that helps reduce thermal heat loss. It helped increase the H.T.F inlet hotness, Reynolds numerical value, and event solar emission. An increase in friction coefficient and Nusselt number was recorded with increased geometric parameters.

A FEM-based resolution method in the form of an inverted triangle, triangle, and semi-circular inserts. The technique was based on a 3-D steady-state and SST k–ω turbulence model that helps reduce thermal stresses. It was recorded that the pressure drop was the highest at the time of semi-circular insertion. Zheng et al. [113] examined that the FVM-based resolution technique based on the 3-D steady-state RNG k–ε turbulence model helped lower the length between the porous rings. The proposed resolution technique ‘Dimple' helps in augmenting heat transfer efficiency. It was analyzed that when the internal diameter of the rings was increased, there was a reduction in the Nusselt number. As a result, the heat transfer performance with porous rings was much higher compared to segmental ring structures. Therefore, it can be said that by introducing FVM based technique, the FEM-based method, and others, there is an improvement in the performance of the P.T.C system, which makes it an economically viable tool for energy production.

## 4 Various applications of solar P.T.C system

In recent times, the availability of fossil fuels has been decreasing consistently, and the rise in its cost is becoming one of the major reasons for finding an alternative energy resource. Hence, SE is gaining prominence in various applications for industrial processes and predominantly in energy generation applications because of its advantages, efficiency, and availability. P.T.C is a line-focusing system that uses different equipment, such as polished metal mirrors and moving parabola reflectors, to collect and process solar radiation onto the focused linear focal line. The solar P.T.C helps to generate heat used for different purposes listed in Table 1.

**Table 1:** Applications of the solar P.T.C systems.

Applications	Description
Electricity generation	Electricity is frequently produced by solar power plants using solar P.T.C systems. The concentrated SE is applied to heat a fluid, which then produces steam to power a turbine and produce electricity. Depending on the location, size, and technology employed, solar power plants using P.T.C systems can produce electricity at a cost between USD 0.20/kWh and USD 0.36/kWh. This is competitive with other ways to produce electricity, such as using fossil fuels or wind energy. Solar power plants can minimize greenhouse gas emissions by replacing fossil fuel-based energy generation and have low operating costs [114,115].
Process heat	Process heat can be produced by solar P.T.C systems for industrial uses such as food processing, chemical manufacturing, and desalination. In Cyprus, the industrial sector is the second-largest fuel consumer, accounting for 60% of all fuel consumption. Using P.T.C systems to generate hot water which can therefore be a potential solution for the industrial sector [116]. Depending on several variables, solar P.T.C systems can produce process heat for as little as USD $0.03 per kWh. This is competitive with alternative process heat sources like biomass and natural gas. By replacing process heat derived from fossil fuels, solar process heat can lower greenhouse gas emissions [114].
Space heating	Space heating for buildings, including residences, educational institutions, and commercial structures, can be accomplished with solar P.T.C systems. SE can be utilized to heat a fluid, which is subsequently circulated through the heating system of a building. To heat a building, Jamadi et al. [117] conducted an experimental comparison of two solar heating systems: the parabolic solar collector (P.T.C) and the flat plate solar collector (FPC). The FPC and P.T.C systems' overall efficiencies were 6 and 12%, respectively. Additionally, it was said that as compared to an FPC heating system, a P.T.C system with less occupied space could produce thermal energy of superior quality. Additionally, it was determined that the P.T.C solar heating system is very suited and effective for use in the building's heating system [117]. Solar P.T.C can provide space heating at a low cost, depending on various factors. This is competitive with other forms of space heating, such as natural gas and electric resistance heating. Solar space heating can reduce greenhouse gas emissions by replacing fossil fuel-based space heating [114].
Solar cooling	Absorption chillers, which can be used to provide cooling for buildings and other uses, can be powered by solar P.T.C systems. The heat produced by the SE is used to power the cooling process. Sulaiman et al. [118] evaluated the effectiveness of a P.T.C-based integrated system for heating, cooling, and electricity generation. Three modes—a solar mode, a solar and storage mode, and a storage mode—are used to conduct their research. According to their findings, the maximum electrical efficiencies for the solar mode, the solar and storage mode, and the storage mode are 15%, 7%, and 6.5%, respectively [118].
Solar drying	The drying of agricultural products like grains, fruits, and vegetables is possible using solar P.T.C systems. The concentrated SE's heated air or liquid can be used to dry the products. Depending on several variables, solar P.T.C systems can be utilized for solar drying for a cost of about USD 0.03/kWh. This is less expensive than alternative drying methods like electric or gas-fired dryers. By substituting solar drying for fossil fuel-based drying, greenhouse gas emissions can be decreased [114,119]. Walz et al. [120] analyzed that P.T.C is an efficient heating device and can be used for portable dryer machines. The system includes a heat sink fin and terminal that automatically allows cross ventilation and the motor fan rotation. It helps generate hot wind waves continuously and acts as a perfect dryer for drying cloth. Thus, it can be said that the P.T.C system is highly efficient in producing heat without using any non-renewable energy and produces heat with simple equipment and techniques cost-effectively.
Solar cooking	Solar P.T.C systems can be managed for cooking, especially in rural areas where access to fuel is limited. Concentrated SE can be utilized to heat a cooking pot or oven. Noman et al. [121] create a simplified mathematical model to assess the performance of a thermally exposed solar parabolic trough cooker. The findings demonstrate that the parabolic trough cooker's maximum water temperature under stagnant conditions was 53.6 °C. Additionally, the observed cooker energy efficiency ranged from 0.11 to 6.5%, and the energy efficiency for direct cooking ranged from 7.6 × 10^−2^ to 2.1 × 10^−2^% [121]. Solar P.T.C systems can be used for solar cooking at a lower cost than other forms of cooking, such as electric or gas-fired stoves. Solar cooking can reduce greenhouse gas emissions by displacing fossil fuel-based cooking.
Water heating	Water can be heated using solar P.T.C systems for household or commercial usage. The heat generated by the sun's radiation is transferred to a fluid, which ultimately warms the water. To produce hot water for large buildings, Bilal et al. [122] investigated the viability of deploying a paired solar P.T.C-latent heat T.E.S system. The system can provide hot water between the ranges of 85–36 °C and 63-38 °C, respectively, during daytime and overnight operation, proving that employing the suggested system with RT-55 as a storage medium is feasible for big buildings [122]. Depending on several variables, solar P.T.C systems can be used to heat water for a cost of about USD 0.03 per kWh. Generally, this is less expensive than other methods of heating water, such as electric or gas-fired water heaters. By substituting solar water heating for fossil fuel-based water heating, greenhouse gas emissions can be decreased [114].
Solar hydrogen production	Solar water splitting is a method that solar P.T.C systems can use to create hydrogen gas. Hydrogen and oxygen are separated from water using concentrated solar radiation. Habibollahzade et al. [123] proposed a new energy system consisting of solar P.T.C, a Rankine cycle, a thermoelectric generator, and a proton exchanger membrane for hydrogen production. The obtained data show that at the most energy-efficient operating conditions, exergy efficiency and total cost are 12.76% and 61.69 $/GJ, respectively, [123]. Solar hydrogen production, a promising method for manufacturing hydrogen without emitting greenhouse gases, is possible with the aid of solar P.T.C systems. The cost of producing hydrogen using SE relies on several variables, including the technology's effectiveness and the price of the raw materials. According to current projections, solar hydrogen production may one day be competitive with other methods of hydrogen production.
Solar desalination	Desalination plants can be powered by solar P.T.C systems, which turn saltwater into freshwater. Using heated fluid by SE can evaporate the seawater. The resulting steam is then condensed to create fresh water. Solar desalination costs vary depending on several variables, including how effective the technique is and how much the materials cost. By replacing desalination using fossil fuels, solar desalination can lower greenhouse gas emissions [124]. Saettone [125] analyzed that the P.T.C system is used in desalination projects to obtain low-cost fresh water. The distiller attached to the P.T.C consists of a solar concentrator and absorber cavity that helps absorb solar radiation in large quantities. The simple thermal insulation and wind protection installed in the cavity absorber help increase freshwater productivity by two folds by efficiently using direction and position during the day. It not only saves the costs related to non-renewable resources but also contributes toward the green and sustainable source of freshwater production.
Solar steam generation	Steam can be produced by solar P.T.C systems for industrial processes like cleaning and sterilizing. Steam is produced by heating a fluid using concentrated SE. Kalogirou et al. [126] investigated the use of a P.T.C system to produce steam. According to the findings, only 48.9% of the solar radiation available is used to generate steam. The remaining amount is lost through thermal or collector losses [126]. By substituting solar steam generation for fossil fuel-based steam generation, greenhouse gas emissions can be reduced.
Solar air conditioning	Adsorption or absorption refrigeration systems that offer to cool for buildings or cars can be powered by solar P.T.C systems. The SE is captured and converted into heat, which powers the cooling process. SE is mainly employed in refrigeration and AC equipment wherein the air temperature decreases below the threshold point by dehumidification and later reheating the air to reduce the temperature and improve the moisture condition. Various researchers have discussed technologies developed for solar thermal cooling and their fundamental efficiencies, which suffer from limitations in design aspects and vapour compression cycles [95].
	When compared to conventional air conditioning systems, the initial installation cost of a solar air conditioning system is somewhat more. However, the usage of renewable energy lowers long-term operating expenses. In addition, tax breaks and subsidies can be accessible for building SE systems. Due to the use of renewable E.Ss rather than fossil fuels, solar air conditioning systems have a substantially lower carbon footprint than conventional air conditioning systems [127].
Solar T.E.S	T.E.S systems can be combined with solar P.T.C systems to store SE for later use. A fluid is heated using the SE that has been captured, and the heated fluid then transmits the heat to a T.E.S medium, like molten salt [128]. To improve the performance of commercial P.T.C concentrated solar thermal power plants with T.E.S capabilities, Praveen et al. [129] developed a fuzzy non-linear programming-based optimization approach employing genetic algorithms. The outcomes demonstrated that the suggested optimization method could produce superior plant efficiency of the order of 16.53% and 17.42% and with a capacity factor greater than 60% when applied to commercial P.T.C plants with T.E.S capability [129]. By storing SE for later use, which can lessen the requirement for E.Ss based on fossil fuels, solar T.E.S devices can aid in lowering carbon emissions.
Solar district heating	For communities or commercial areas, district heating can be provided using solar P.T.C systems. The heated fluid is then circulated through a system of pipes to heat buildings and other users using the SE that has been captured using P.T.C. Tian et al. [130] employed the TRNSYS-GenOpt model to optimize the important design elements of the plant, such as the locations of both types of collectors, the amount of storage, the orientation of the P.T.Cs, and other factors. The lowest net levelized cost of heat for hybrid solar heating plants was discovered to be around 0.36 DKK/kWh. Solar collectors can be used in the district heating network in this study to reduce the system-levelized cost of heat by 5–9% [130]. Solar district heating systems can help reduce carbon emissions by using renewable energy instead of traditional E.Ss.
Solar drying of sewage sludge	Before disposal or reuse, sewage sludge can be dried using solar P.T.C devices. The sludge is heated by concentrated SE, which causes it to dry up and lose volume. As less sewage sludge needs to be disposed of, solar drying of sludge can result in cost savings. By eliminating the need for fossil fuel-based drying techniques, solar drying of sewage sludge can assist sewage treatment plants in lowering their carbon footprint.
	Eldredge [131] uses SE to pyrolyze sewer sludge using solar P.T.C. The findings of this study indicate that it is theoretically viable to use an evacuated tube P.T.C to perform solar-aided pyrolysis of sewer sludge utilizing steam as the pyrolysis medium. The findings demonstrate that steam offers thermal advantages over either N_2_ or CO_2_ as the pyrolysis medium, improving the P.T.C reactor's thermal efficiency and pyrolysis temperatures [131].
Solar process heat for mining	Solar P.T.C systems can produce process heat for mining activities like ore processing and smelting. To learn more about the technical, financial, and emissions performance of solar heating systems for the biggest copper mining operations in Chile, Quiones et al. [132] studied the integration of solar heating into the copper refining process. According to the economic study, solar heating technologies are a viable alternative for reducing costs and emissions in copper mining at the current price of fossil fuels [132]. Solar process heat for mining operations can help reduce carbon emissions by using renewable energy instead of fossil fuels [114].
Solar-assisted steam-enhanced oil recovery	Steam can be produced by solar P.T.C systems in oil fields for steam-enhanced oil recovery. To increase oil production, SE is used to heat a fluid, which creates steam that is then injected into the oil reservoir. For improved oil recovery, Gajadhar et al. [133] examined the design and use of a solar P.T.C in conjunction with a heat exchanger. It was determined that the parabolic troughs and heat exchanger tubes would cost USD 119,562 in total. By using a mass flow rate of 46 kg/s for the water in the heat exchanger, and maintaining a maximum steam-oil ratio of 4.5, around 1630 barrels of oil were profitably produced after one day of steam injection [133].
Sterilizing	Medical equipment, laboratory equipment, and other goods that need to be sterilized can all be sterilized with solar P.T.C systems. The device warms water or steam to a high temperature, which destroys germs and other microbes. In hospitals, laboratories, and other healthcare settings, this procedure is frequently employed. Using P.T.C, Abdel Dayem et al. **[**127**]** investigated the thermal and optical performance of solar water disinfection systems experimentally and numerically. The study considers visual, thermal, thermal after-visual, and combined disinfection. From a biological standpoint, the system with the thermal effect that comes after the visual effect has the cleanest water. Solar sterilization systems reduce reliance on non-renewable E.Ss, leading to a reduction in greenhouse gas emissions and overall carbon footprint [134].
Pasteurizing	Food and beverage goods can be pasteurized using solar P.T.C systems. A liquid or food product is heated to a specified temperature for a predetermined amount of time by the system, which eliminates unwanted germs and microbes while keeping the product's quality and flavour. The dairy, juice, and beer industries frequently use this application.
	Bigoni et al. [135] investigated the efficacy of solar radiation-based water pasteurization using a P.T.C system. They discovered that aggregated heat from a parabolic trough was able to inactivate 9 log10 of *E. coli* and other germs, but that bacterial regrowth resumed after 72 h at 30 °C.
	In addition to improving pasteurization efficiency and lowering energy costs, pasteurization with solar P.T.C systems can also have a positive financial impact. Minimizing the usage of fossil fuels and the associated greenhouse gas emissions, it can also benefit the environment. Lowering the chance of contaminating food and beverage goods with hazardous germs and microbes can also increase the safety and quality of those products [135].
Degreasing	Another application for solar P.T.C systems is degreasing machinery and industrial equipment. Grease and other impurities can be dissolved and removed from surfaces by the high temperature of the steam or water, leaving them clean and ready for use. This technology is frequently used in the industrial and auto industries. When compared to conventional degreasing techniques that use fossil fuels, degreasing solar P.T.C systems can save money by eliminating the need for pricy, hazardous chemicals and by using less energy. Additionally, it can benefit the environment by minimizing the quantity of harmful waste produced by conventional degreasing techniques [136].

## 5 An economic potential gap in P.T.C

While P.T.C technology has been shown to have significant potential for generating clean energy, several economic factors can limit its widespread adoption. When the conversion of SE to thermal energy takes place, there is a reduction in the performance of P.T.C owing to heat loss at high operating temperatures. It exerts adverse implications on the performance capabilities of the P.T.C as there is a reduction in power generation, high consumption of resources, and an increase in cost. Moreover, it analyzes the economic costs associated with constructing a solar power plant with the help of P.T.C. As per the analysis, it was found that different cost is associated with establishing a solar power plant and extracting energy from it. It includes costs such as initial costs, system costs, land costs, mainland costs, operation and maintenance costs, and loan interest costs. For many potential users, this can be a substantial obstacle, especially in areas with low energy costs or with few renewable energy subsidies. P.T.C technology's economic potential may also be constrained by the fact that it may be less efficient than some other varieties of solar technology. Despite these obstacles, a variety of tactics can be employed to raise the economic potential of P.T.C technology. For instance, by enhancing these systems' efficiency and lowering their prices through technological advancement and economies of scale, they could become more competitive with traditional E.Ss. Policies like tax breaks, financial aid, and feed-in tariffs can also contribute to increasing the economic attraction of P.T.C technology for users.

In China, to establish a solar power plant, the major cost was related to the attainment of land for erecting the plant. The system cost ranged between $2.6/W and $11.27/W for P.T.Cs that did not have a storage facility and ranged between $7.3/W to $11.3/W for P.T.Cs that had storage facilities. For example, the efficacy of the power plant in China was analyzed by considering different aspects such as annual costs, discount rate, initial costs, level of solar irradiation, and technology. As per the analysis, it was found that the plants that functioned with the help of P.T.C were efficient and produced power in terms of the L.C.O.E.

Bishoyi & Sudhakar [137] analyzed that a P.T.C system-based power plant was economically viable in locations with DNI over 1800 kWh/m^2^/year. For example, different locations in India such as Tamil Nadu, Coimbatore, Gujarat, Palanpur, Karnataka, Udaipur, Tumkur, Rajasthan, and others have more than 1800 kW h/m^2^/year. Due to high DNI levels, there is an increase in heat extraction from solar radiation, which augments the power generation capacity of solar power plants. It helps in reducing the consumption of fossil fuels and increasing the use of renewable sources of energy in power generation, which makes P.T.C economically viable for the production of power.

A study was conducted by Taylan et al. [138] to compare the economic viability of P.T.C and PV systems of power generation. In the PV system, SE is converted into DC energy using inverters. The inverters are connected to single-phase AC electricity and a transformer that helps generate electricity through input simulations. On the other hand, in P.T.C, parabolic shape reflectors are used to reflect sunrays, and H.T.F absorbers extract heat from the sun's rays. It also includes an MCRT/FVM-based resolution technique that helps reduce thermal heat loss and increase the H.T.F inlet temperature level. A comparison analysis has been shown in Table 2 that determines the financial aspects of both techniques.

**Table 2:** Parameters used in the economical viability simulations by Taylan et al. [138].

Parameter	Value for P.T.C	Value for PV
Capital cost ($/kW)	6065	1745
Fixed operating cost ($/kW)	66	14
Variable operating cost ($/kWh)	0.0040	0
Analysis period (years)	25	25
Inflation rate (%/year)	15	15
Interest rate (%/year)	8	8

According to Table 2, various financial parameters such as variable operating cost, fixed operating cost, capital cost, and analysis period are used to determine the financial aspects of the P.T.C and PV technique. Based on the value of the various components of P.T.C and PV and their performance, it was discovered that P.T.C demonstrated greater efficacy and economic viability than the PV technique, as shown in Table 3. According to Table 3, the P.T.C production capacity in the annual energy segment was 6.30 GWh, and the PV plant production capacity was 4.95 GWh. P.T.C plant performance was 89.5% in the gross-to-net conversion segment, while PV plant performance was 81%. As a result, the P.T.C plant outperformed the PV plant in terms of efficacy and economic viability.

**Table 3:** Summary of comparison of 3 MW P T.C and PV power plants reported by Taylan et al. [138].

Parameter	P.T.C Plant	PV Plant
Annual energy generation	6.30 GWh	4.95 GWh
Gross-to-net conversion	89.5%	81%
Capacity factor	24%	18.9%
Annual water usage	1.8 m^3^	–
Levelized COE	14.7 cents/kWh	4.94 cents/kWh

The introduction of a stationary concentrator in the P.T.C helps in reducing the cost related to the operation of the system. Moreover, a significant reduction in the operating cost and an increase in the performance of P.T.C was also recorded when there was the installation of insulation inside the absorber tube. Due to installing of insulation inside the absorber tube, there was a reduction in the receiver cost by 20% in comparison to the cost of the conventional P.T.C system. Yang et al. [139] conducted a study to analyze the economic potential of P.T.C and found that when a double-coating absorber tube was introduced in the conventional P.T.C system, there was a reduction of 5% in L.C.O.E. On the other hand, when the nanofluid-oriented P.T.C was included in the conventional P.T.C workings, there was a reduction in the L.C.O.E by 1%. Bellos & Tzivanidis et al. [140] analyzed thee helical fins which were fixed on the interior lead surface of the conventional P.T.C system, and there was an increase in the plant electricity production capacity by 2% with a slight increase in the investment cost by 0.5%. In the study by Karathanassis et al. [141], it was found that when a parabolic concentrator was used along with the flattened PVT receiver, it helped produce power cost-effectively by concentrating on solar antagonists. As per the previous data, it can be said that there have been studies related to the estimation of the economic potential of P.T.Cs. However, scholars have not discussed these financial aspects related to solar to thermal conversion in the P.T.C segment. As a result, it created a gap and lack of sufficient information on the economic potential of P.T.C. Therefore, the current research highly focuses on analyzing the economic potential of P.T.C and its technical aspect so that the gap between previous and current literature eliminates.

According to the International Renewable Energy Agency's (IRENA) 2020 report, the global weighted average L.C.O.E for P.T.C was 0.185 $/kWh in 2018, which may be greater than the L.C.O.E of some conventional E.Ss like natural gas power plants, the real value of the location and operational circumstances determine the L.C.O.E for P.T.C. The cost of P.T.C technology has, however, significantly decreased because of recent technological breakthroughs and economies of scale [142].

According to Sajed Sadati et al. [143], the L.C.O.E for P.T.C systems without storage is found to be 0.27 USD/kWh, 0.226 USD/kWh for P.T.C systems with 7.5 h of storage, and 0.192 USD/kWh for PV systems. Given that it considers both the time value of money and investment risks, the discount rate is one of the crucial variables in financial research. The International Energy Agency (IEA) has provided conservative discount rate assumptions of 10%–12% for PV systems and 10%–15% for C.S.P systems (including P.T.C).

A C.S.P plant's capital expenses were estimated to range from 3000 to 5067 $/kW in 2012. The IEA predicts that the capital cost of a C.S.P plant will be roughly 4200 $/kW in 2014 and 3000 $/kW in 2020. Like this, a 2013 report from the IRENA estimated that installed parabolic trough systems cost between 3400 and 4600 $/kW for load factors of 20%–27% and projected a 30%–50% drop in capital costs by 2020 as a result of technological advancement and economies of scale brought on by the expanding use of C.S.Ps [144].

The IRENA reports that the total installed C.S.P capacity growth worldwide reached about 5.5 GW at the end of 2018 (4.3 times what it was in 2010). Similarly, the 0.5 GW of capacity built in 2018 had a global weighted average L.C.O.E of US$ 0.185/kWh, which is 26% less than in 2017 and 46% less than in 2010. With this decrease in electricity costs (26%) in 2018, C.S.P was placed #1, followed by biofuel (14%), solar PV and onshore wind (13%), hydropower (11%), geothermal energy (1%), and offshore wind (1%) [145].

The L.C.O.E of electricity generated by conventional E.Ss like coal and natural gas is influenced by the plant's location and type. However, according to the International Energy Agency's (IEA) most recent “World Energy Outlook 2021″ report, coal-fired power plants have an average L.C.O.E of around 5 to 7 cents per kWh, and natural gas-fired power plants have an L.C.O.E of roughly 4 to 6 cents per kWh [146].

Therefore, even though the cost of P.T.C technology has been falling in recent years, there is still a potential economic difference between P.T.C and conventional E.Ss. Research and development activities are concentrated on enhancing the effectiveness and dependability of the technology as well as lowering the cost of materials and components to further lower the L.C.O.E of P.T.C.

## 6 Life cycle assessment of P.T.C over conventional E.Ss

A life cycle assessment is an approach for determining how a product, process, or service will affect the environment throughout its entire life cycle [128]. In this section, we will examine the L.C.As of different energy generation technologies, including coal, natural gas, nuclear power plants, biofuels, P.T.Cs, solar PV, hydropower from reservoirs and rivers, ocean, and wind energy. L.C.A can be used to analyze the environmental advantages and disadvantages of adopting P.T.C technology for energy generation vs. other E.Ss. L.C.A is a methodology for assessing the environmental impacts of any product, process, or service at various stages of its life cycle. This analysis will help us understand the need for scaling up a specific technology among them by informing us about the various harmful gases and bio-products emissions during these technologies' various life cycle stages. It should be noted that the environmental impacts of P.T.C can vary depending on several factors, including the location and size of the plant, the materials and components used, and the disposal method for end-of-life components. In addition, some environmental impacts, such as land use and water consumption, can be significant for P.T.C and should be carefully considered in the L.C.A [146].

**Fig. 8:**
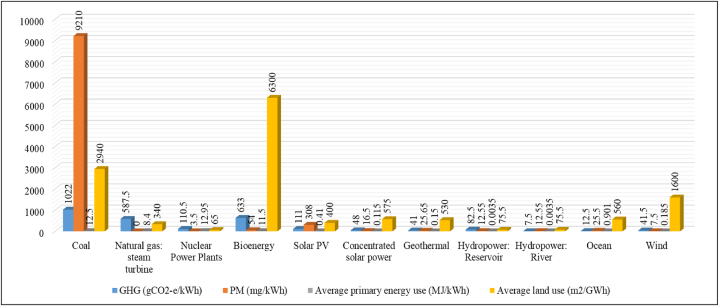
Graph indicating the emissions generated by different technologies.

In this direction, using existing literature, a comparative analysis is conducted among various technologies based on various environmental impacts such as greenhouse gas (G.H.G), particulate matter, average primary energy use, and average land use. As shown in Fig. 8, P.T.C, a concentrated solar power (C.S.P) component, is also compared to other technologies [[147], [148], [149], [150], [151], [152], [153], [154], [155], [156], [157], [158], [159], [160], [161], [162], [163], [164], [165], [166], [167], [168], [169], [170], [171], [172]].

According to this analysis, among the various technologies, geothermal, ocean, and C.S.P parabolic trough plants (P.T.C) emit less waste and harmful gases on average of 2.16%, 2.23%, and 2.39%, respectively, over their lifetime. Thus, it demonstrates the P.T.C technique's superiority over other energy generation techniques.

Fig. 8 shows the emissions generated by different technologies. The emitted G.H.G for coal, natural gas steam turbine, nuclear power plants, bioenergy, solar PV, concentrated solar power, geothermal, hydropower reservoir, hydropower river, ocean, and wind are 1022, 587.5, 110.5, 633, 111, 48, 41, 82.5, 7.5, 12.5, and 41.5 gCO_2_-e/kWh, respectively. This data provides information about the environmental impact of different methods of generating G.H.G. Climate change and global warming are caused by the presence of greenhouse gases, such as carbon dioxide. The burning of fossil fuels, such as coal and natural gas, releases large amounts of greenhouse gases into the atmosphere. Therefore, it is important to know the amount of greenhouse gas emissions associated with different methods of generating electricity so that we can make informed decisions about which sources to use. This information can be used to compare the environmental impact of different E.Ss and help us to choose cleaner, more sustainable options for generating electricity.

According to a 2020 IEA report, solar thermal systems like P.T.C generally have fewer life cycle environmental consequences than fossil fuel-based technologies in terms of criterion pollutants, including nitrogen oxides (NO_x_), sulphur dioxide (SO_2_), and particulate matter (PM). The paper points out that solar thermal technologies still have some negative environmental effects, including those related to land use and water use [173,174].

Furthermore, Burkhardt et al. [175] investigated the L.C.A study of a 103 MW parabolic trough C.S.P in Daggett, California, USA, along four important measures, namely, G.H.G emissions, cumulative energy demand (CED), water consumption, and the energy payback time (EPBT), to evaluate design alternatives with a reference design for capacity expansion. Dry cooling, a thermocline TES, and a synthetically derived nitrate salt were chosen as design alternatives. It was discovered that the reference C.S.P emits 26 g CO_2_ eq per kWh and consumes 4.7 L per kWh of water with approximately one year of EPBT. Other design options, such as dry cooling, reduce water consumption by 77% while increasing G.H.G emissions and CED by 8%. Compared to naturally mined nitrate salts, the use of synthetic nitrate salts results in a 52% increase in greenhouse gas emissions. Furthermore, switching from two tanks to a thermocline TES design reduces overall G.H.G emissions from salts.

In another study, Burkhardt et al. [176] used consistent performance parameters and plant design, system boundaries, and global warming potential to reduce variabilities and concluded that the G.H.G range for solar-only and non-hybrid T.E.S plants is 20–34 kg CO_2_ eq/MWh.

Corona et al. [177] investigated the C.S.P using the parabolic trough having net efficiency of 16%. This practice significantly impacts the environment, and more research is needed to determine its long-term viability. The effects of combining this practice with natural gas have also been investigated. The same inventory was gathered for a commercial wet-cooled 50 MW C.S.P plant based on parabolic troughs, which gives an environmental profile as climate change of 26.6 kg CO_2_ eq/KWh, marine ecotoxicity 276 g 1,4-DB eq/KWh, human toxicity 13.1 kg 1,4-DB eq/KWh, natural land transformation 0.005 m^2^/KWh, acidification 166 g SO_2_ eq/KWh and eutrophication 10.1 g P eq/kWh. This study also concluded that the hybridization of natural gas significantly deteriorated environmental performance.

Studies have generally indicated that P.T.C technology can be less harmful to the environment than traditional E.Ss like fossil fuels. According to a 2019 NREL study, P.T.C technology, as opposed to natural gas combined cycle power plants, had lower life cycle greenhouse gas emissions intensity. The materials used to make the systems, the E.Ss utilized to create those materials, the location and use of the systems, and other factors can all have an impact on the environment. In areas with intense solar irradiation, for instance, P.T.C systems may have a lesser environmental impact than those with low solar irradiation because they can produce more energy.

Research has shown that Parabolic trough C.S.P has very little global warming potential and other environmental impacts compared to fossil fuels, nuclear power, and some renewable E.Ss such as solar and hydro by the reservoir. With higher efficiencies of the system, the environmental performance will further improve. Hence, this technology will likely improve significantly in the future.

## 7 Discussion

The section discusses energy and energy performance, computational fluid dynamics analysis, performance enhancement techniques, thermal analysis, economic and environmental performance, and life cycle comparison of P.T.Cs to conventional E.Ss.

### 7.1 Energy and energy performance

Our analysis revealed that P.T.Cs have tremendous potential for efficiently converting solar radiation into thermal energy. Thermal efficiency, or the ratio of thermal energy captured by the collector to the SE incident on the collector, is commonly used to assess the energy performance of P.T.C. The quality of the reflector, the layout of the receiver tube, and the operating conditions can all affect the thermal efficiency of P.T.C, which can range from 50% to 60%. Because the thermal energy produced by the collector is of lesser quality than the SE that is reflected on the collector, the exergy efficiency of P.T.C is often lower than its thermal efficiency. This is because some of the energies of the solar radiation are lost due to the temperature difference between the focused solar radiation and the heat transfer fluid in the receiver tube. The heat transfer process' irreversibility is the cause of P.T.Cs' low energy efficiency.

This review article provides the various analytical approaches used to evaluate the thermal performance and exergetic performance of P.T.Cs, which can help improve efficiency and reduce the cost of electricity generation. Analyzing H.T.F temperature, mass flow rate, and solar radiation is necessary to evaluate the performance of dynamics present in P.T.C. The exergetic performance of P.T.Cs can be determined by assessing the pressure loss and effective thermal input in solar P.T.Cs. The effective thermal input in solar P.T.Cs is the primary element affecting the performance and efficiency of P.T.Cs. The formation of entropy and absorbed solar radiation exergy by the solar receiver tube and reflector can be determined using the Petela model, which helps calculate the absorber tube's exergy rate. The P.T.Coffers several potential benefits, including.

1. Renewable energy: P.T.Cs generate electricity using renewable energy from the sun, which can help reduce reliance on fossil fuels and mitigate climate change.
2. Cost-Effective: P.T.Cs are a cost-effective option for generating electricity in areas with high levels of direct solar radiation. The technology has a proven track record of commercial viability and operational reliability.
3. Scalable: P.T.Cs can be easily scaled up or down depending on the energy demand, making them suitable for a wide range of applications.
4. Modularity: P.T.Cs are modular in design, which allows for easy installation and maintenance.

However, there are some drawbacks that limit the widespread adoption of the technology, including.

1. Land use: P.T.Cs require large land areas to achieve economies of scale, which can be a challenge, especially in densely populated areas.
2. Water consumption: P.T.Cs require water for cooling and cleaning, which can be a scarce resource in some regions.
3. Energy storage: P.T.Cs require energy storage systems to provide electricity when the sun is not shining, which can add to the overall cost of the technology.
4. Environmental impact: P.T.Cs can have environmental impacts, such as land use changes, habitat fragmentation, and visual impacts.

### 7.2 Computational fluid dynamics (CFD) analysis

The analysis of the P.T.Cs has been facilitated by the use of CFD, a computer-based simulation technique that allows for the study and resolution of issues involving fluid flows, heat transport, and related processes. Widely used in various industries, including aerospace, automotive, energy, and environmental engineering. CFD has several potential benefits when applied to the analysis of P.T.Cs. For example, CFD simulations can optimize the design of P.T.Cs, improving their efficiency and reducing costs while also allowing for the study of fluid behavior in varying conditions, such as wind speeds, to improve the reliability of P.T.C systems. Additionally, the use of CFD in P.T.C analysis can reduce the need for costly and time-consuming experimental testing, as detailed information about fluid behavior can provide information about the design of future experiments and reduce overall testing expenses.

In the context of P.T.Cs, CFD is used to simulate, evaluate, and optimize P.T.C engineering ideas, resolving issues with fluid mechanics and heat transfer design and providing significant insights into replicating data, flow fields, and design processes. CFD also allows for the assessment of the thermal-hydraulic performance of the H.C.E and the examination of the surrounding wind conditions. The use of actual boundary conditions in predicting the efficacy of P.T.Cs is crucial, with heat flux being an important aspect related to the H.C.E absorber performance analysis. CFD analysis can also include radiation models and heat transfer mechanisms to analyze annulus conditions, with the use of ANSYS Fluent software being particularly helpful in modeling the geometric structure of P.T.Cs.

One of the key advantages of CFD in P.T.C analysis is its ability to provide detailed information about flow fields, which helps identify areas of flow disturbances and their impact on system performance. This information can be used to make design modifications or adjustments to improve overall performance. CFD can also be used to evaluate the impact of different materials on P.T.C performance, with researchers using CFD simulations to assess the thermal properties of materials and determine which are best suited for use in P.T.C systems. So, the use of CFD in P.T.C analysis has the potential to improve efficiency and reduce the cost of electricity generation. However, the CFD modeling is complicated by the complicated geometry and fluid flow in P.T.Cs. Therefore, additional study is required to create CFD models for P.T.Cs that are more precise and effective.

### 7.3 Performance enhancement techniques

The review discusses performance enhancement techniques that are currently being researched and developed to improve the efficiency, cost-effectiveness, and reliability of P.T.Cs utilized in C.S.P systems. It has been suggested that using hybrid systems, advanced control systems, novel materials (such as nanofluids), heat pipes, and reflecting coatings will all increase the performance of P.T.Cs.

One of the critical challenges faced by P.T.Cs is the intermittent and unpredictable nature of solar radiation, which can impact the system's ability to generate electricity continuously. T.E.S is an essential component of C.S.P systems, enabling the storage of solar heat during the day and using it to generate electricity during periods of low sunshine. Researchers are exploring the use of novel materials with higher heat capacity, such as phase-change materials, to improve storage capacity and affordability. They are also developing sophisticated control systems that use machine learning and predictive algorithms to optimize the charging and discharging of T.E.S systems based on real-time weather data and electricity demand forecasts.

Hybrid systems that combine P.T.Cs with other renewable energy technologies such as photovoltaics, wind power, or geothermal energy are also being investigated to maximize energy production and boost system reliability. For instance, photovoltaic systems can be used to supplement the electricity generation of P.T.Cs, while wind power systems can be utilized to enhance P.T.Cs' ability to produce electricity by collecting the kinetic energy of the wind. Researchers are also exploring the use of cutting-edge control systems to enhance the upkeep and repair of P.T.Cs, including the monitoring and diagnosing of system issues using sensors and other technologies.

Another critical area of research is the use of novel materials to improve the effectiveness and affordability of P.T.Cs. Researchers are exploring the use of nanostructured materials, selective coatings, and iron-based alloys to enhance the absorption and reflection of sunlight and reduce costs. They are also investigating the use of lightweight components to make the construction of the supporting structure of P.T.Cs more affordable. However, there are challenges associated with the introduction of novel materials, such as durability, heat resistance, and manufacturing costs. To better understand the P.T.C's commercial viability and long-term durability, more study is required.

### 7.4 Thermal analysis of P.T.Cs

Understanding the behaviour of P.T.Cs under various operating situations requires thermal analysis. The thermal efficiency of P.T.Cs, which is a measure of how effectively P.T.Cs convert solar radiation into thermal energy, and the various factors that can impact it. One of the main benefits of P.T.Cs is that they are a proven technology with a long operating history, and their performance and reliability have been well-established. They are also a relatively mature technology with a well-established supply chain for components and systems. Additionally, P.T.Cs are a flexible technology that can be used for both electricity generation and process heat applications, making them suitable for a variety of industrial and commercial applications.

The thermal analysis of P.T.Cs helps estimate the temperature at the surface level, determine the efficiency of the solar absorber, and evaluate the thermal loss of the H.C.E. Thermal models are developed to determine the temperature profiles and heat flux in thermal analysis. The accurate thermal performance of H.C.E is determined by equation (14), which helps determine the local heat flux allocation along the circumference of the H.C.E.

The thermal efficiency of P.T.Cs is affected by several factors, such as the collector's design, the quality and type of the H.T.F, and the system's operating conditions and parameters. There are various techniques and approaches, such as ray tracing and transient modeling, that can be used to analyze the optical and flow features of P.T.Cs and improve their overall efficiency. Further study is required to determine how external elements like wind and dust affect the thermal behaviour of P.T.Cs.

### 7.5 Economic and environmental performance

The use of P.T.C for solar power generation has significant potential for generating clean energy. However, there are economic factors that can limit its widespread adoption. One of the main drawbacks is the reduction in the performance of P.T.C owing to heat loss at high operating temperatures, which can result in a decrease in power generation, high consumption of resources, and an increase in cost. Additionally, the cost of establishing a solar power plant with the help of P.T.C includes various costs such as initial costs, system costs, land costs, mainland costs, operation and maintenance costs, and loan interest costs, which can be a substantial obstacle, especially in areas with low energy costs or with few renewable energy subsidies.

To increase the economic potential of P.T.C, various tactics can be employed, such as enhancing the technology's efficiency and lowering its prices through technological advancement and economies of scale. Policies like tax breaks, financial aid, and feed-in tariffs can also contribute to increasing the economic attraction of P.T.C technology for users. Studies have shown that P.T.C technology can be economically viable in locations with high DNI levels, which are areas that have more than 1800 kW h/m^2^/year.

Several studies have been conducted to estimate the economic potential of P.T.Cs, and recent technological breakthroughs and economies of scale have significantly reduced the cost of P.T.C technology. However, research and development activities are concentrated on enhancing the effectiveness and dependability of the technology as well as lowering the cost of materials and components to further lower the L.C.O.E of P.T.C. Therefore, additional research is required to evaluate P.T.Cs' economic and environmental performance in various scenarios and to contrast it with that of other renewable and conventional E.Ss.

### 7.6 Life cycle assessment (L.C.A) of P.T.Cs over conventional E.Ss

L.C.A is a crucial technique for assessing the long-term environmental effects of P.T.Cs. The L.C.A of energy generation technologies is essential for understanding their environmental impacts and informing decisions about which sources of energy to use. The manuscript's section on the L.C.A of P.T.C technology compared to other E.Ss provides valuable insights into the environmental advantages of P.T.C technology. The analysis shows that P.T.C technology emits fewer harmful gases and waste over its lifetime compared to other energy generation technologies, including coal, natural gas, nuclear power plants, biofuels, solar PV, hydropower from reservoirs and rivers, ocean, and wind energy.

The environmental impact of P.T.C technology can vary depending on several factors, including the location and size of the plant, the materials and components used, and the disposal method for end-of-life components. The study also highlights the environmental impacts of land use and water consumption associated with P.T.C technology, which should be carefully considered in the L.C.A.

Several studies have investigated the environmental impacts of P.T.C technology, including G.H.G emissions, cumulative energy demand, water consumption, and energy payback time. These studies indicate that P.T.C technology has lower G.H.G emissions compared to fossil fuel-based technologies in terms of criterion pollutants, such as SO_2_, NO_x_, and PM. However, P.T.C technology still has negative environmental effects, including those related to land use and water use. To evaluate the environmental impact of P.T.Cs in various circumstances and to determine their sustainability throughout the course of their life cycles, more research is still required.

## 8 Critical hints for future steps in the field

Our review identified several critical hints for future steps in the field of P.T.Cs. These include creating more precise and effective CFD models, examining the viability and durability of performance-enhancing approaches from an economic perspective, and examining the impact of ambient variables on the thermal behaviour of P.T.C.

Constructing a superhydrophilic or superhydrophobic coat on the P.T.C may be useful in future research, depending on the specific application and desired outcome. A superhydrophilic coating would be highly wettable and would allow for rapid and complete wetting of the P.T.C surface, which could be beneficial for applications that require rapid heat transfer or efficient energy conversion. For example, in some heating or cooling systems, a superhydrophilic coating could enhance heat transfer by promoting better contact between the P.T.C surface and the working fluid. On the other hand, a superhydrophobic coating would repel water and other liquids, making the P.T.C surface highly non-wetting. This could be useful in applications where it is important to keep the P.T.C surface clean and free from contamination, such as in medical or laboratory equipment. Additionally, a superhydrophobic coating could provide protection against corrosion or damage from moisture.

The P.T.C's surface area will expand with either superhydrophilic or superhydrophobic coatings, which will enhance the amount of solar radiation the collector can absorb, which could result in higher temperatures and more energy output. The receiver tube, which is typically placed along the focal line of the parabolic mirror, receives more sunlight when it is concentrated in a larger area.

## 9 Conclusions

P.T.C extracts SE for industrial and household purposes, such as pasteurization, drying, etc. It is an efficient method of utilizing SE and converting it into thermal energy to perform different functions such as pressing, degreasing (textile industry), and many more. However, the major issue with the use of the P.T.C is its limited use of SE, which is not a continuous E.S. Therefore, optical and thermal analysis modifications were introduced in the conventional P.T.C to increase its productivity and performance. It was found that the performance of enhancement techniques of P.T.Cs, such as novel designs, passive heat transfer improvement, and nanoparticle-laden fluid flow. It was also analyzed that the thermal insulation and wind protection installed in the cavity absorber helped increase the freshwater productivity levels by two folds by efficiently using direction and position during the day. It saved the costs of non-renewable resources, contributed toward green, and generated a sustainable E.S. Therefore, it can be said that P.T.C is an efficient system that helps in generating heat and power by using simple techniques and affordable equipment, which makes it economically viable.

P.T.C is very useful in applications where energy is required in the form of heat. When it comes to electrical generation, it is currently not a financially viable option. Further research is needed to improve the efficiency of the P.T.C by implementing or modifying existing techniques to compete with other sources and make P.T.C an economically competitive option. Aside from P.T.C, improving the performance of the system's components will aid in further improving overall efficiency and environmental performance, lowering energy production costs.

A P.T.C's levelized cost of electricity (L.C.O.E) should be as low as possible to increase its long-term economic viability. The L.C.O.E was reduced by 1% when the nanofluid-oriented P.T.C was incorporated into the standard P.T.C workings. The emitted greenhouse gases for coal, natural gas steam turbine, nuclear power plants, bioenergy, solar PV, concentrated solar power, geothermal, hydropower reservoir, hydropower river, ocean, and wind are 1022, 587.5, 110.5, 633, 111, 48, 41, 82.5, 7.5, 12.5, and 41.5 gCO_2_-e/kWh, respectively. This information can be used to compare the environmental impact of different E.Ss and help us to choose cleaner, more sustainable options for generating electricity.

Furthermore, more research is needed to address issues in energy storage technology, as SE does not last all day. The high initial manufacturing cost of solar thermal systems must also be addressed.

## Author contribution statement

All authors listed have significantly contributed to the development and the writing of this article.

## Data availability statement

Data will be made available on request.

## Declaration of competing interest

The authors declare that they have no known competing financial interests or personal relationships that could have appeared to influence the work reported in this paper.

## Abbreviations

AC: Alternative current (A)
CFD: Computational fluid dynamics
C.S.P: Concentrated solar power
CED: Cumulative energy demand (MJ/kg)
DNI: Direct Normal Irradiance (W/m^2^)
DSG: Direct steam generation
DO: Discrete ordinate
EPBT: Energy payback time (years)
E.S: Energy source
FDM: Finite difference method
FEM: Finite element method
FVM: Finite volume method
FVM: Finite volume method
G.H.G: Greenhouse gas (gCO_2_-e/kwh)
H.C.E: Heat collector element
H.T.F: Heat transfer fluid
INR: Indian Rupee
IEA: International Energy Agency
L.C.O.E: Levelized cost of electricity
L.C.A: Life cycle assessment
MCRT: Monte carlo ray tracing
NREL: National Renewable Energy Laboratory
1-D: One-dimensional
P.T.C: Parabolic trough collector
PM: Particulate matter
PV: Photovoltaic
PVT: Photovoltaic-thermal
IRENA: Renewable Energy Agency
RNG _k–ε_: Reynolds-averaged Navier-Stokes k-epsilon
S2S: Surface to surface
SE: Solar energy
T.E.S: Thermal energy storage (J or Wh)
T.E.S.S: Thermal energy storage system
LCR: Thermal performance of H.C.E

**Symbols**
*A*_ro_: Absorber area (m^2^)
Ex_u_: Absorber tube's exergy rate (J/s)
W: Aperture (m^2^), Width (m)
D: Diameter (m)
*S*: Entropy (J/^o^C)
*f*: Focal distance (m)
C: Geometrical concentration ratio, specific heat capacity (J/Kg.K)
Q: Heat (J)
*I*: Hourly irradiation (J/m^2^.h)
K: Incidence-angle modifier, thermal conductivity (W/m.K)
L: Length (m)
r: Reflectance
m: The mass flow rate of the fluid (kg/s)
n: The shape factor
T: The temperature (^o^C or K)
DT: The temperature difference between the inlet and outlet of the collector (^o^C or K)
U_L_: Thermal loss coefficient (W/(m^2^·K))
*A*_a_: Total collector aperture (m^2^)
W_a_: Width of the absorber tube (m)

**Greek symbols**
α: Absorber surface (m^2^)
θ: Angle (angle between surface and incident radiation) (^o^)
η: Efficiency
Г: End loss factor
τ: Glass envelope (m)
*θs*: Half-angle subtended by the sun (^o^)
ρ: Reflectivity, density (kg/m^3^)
*φ*_*r*_: Rim angle (^o^)
γ: The energy incident levels (W/m^2^)
μ: The viscosity (Pa.s)
φ: The volume fraction of nanoparticles

**Subscript**
a: Ambient, aperture
0: Before irradiation
D: Diffuse
f: Fluid
gen: Generated
i: Incident
*n*: normal
o: Overall, out, optical
r: Receiver tube
S: Surface, sun
b: The base fluid, beam
in: The inlet
*nf*: The nanofluids
p: The nanoparticle
out: The outlet
S: The sun
th: Thermal
U: Useful
